# Eosinophil-derived chemokine (hCCL15/23, mCCL6) interacts with CCR1 to promote eosinophilic airway inflammation

**DOI:** 10.1038/s41392-021-00482-x

**Published:** 2021-02-28

**Authors:** Xufei Du, Fei Li, Chao Zhang, Na Li, Huaqiong Huang, Zhehua Shao, Min Zhang, Xueqin Zhan, Yicheng He, Zhenyu Ju, Wen Li, Zhihua Chen, Songmin Ying, Huahao Shen

**Affiliations:** 1 Key Laboratory of Respiratory Disease of Zhejiang Province, Department of Respiratory and Critical Care Medicine, the Second Affiliated Hospital of Zhejiang University School of Medicine, Hangzhou, 310009 China; 2 Department of Anatomy, Zhejiang University School of Medicine, Hangzhou, 310058 China; 3 Key Laboratory of Regenerative Medicine of Ministry of Education, Institute of Aging and Regenerative Medicine, Jinan University, Guangzhou, Guangdong 510632 China; 4 International Institutes of Medicine, Zhejiang University School of Medicine, Yiwu, 322000 China; 5 Department of Pharmacology, Zhejiang University School of Medicine, Hangzhou, 310058 China; 6 State Key Lab of Respiratory Disease, Guangzhou, 510120 China

**Keywords:** Inflammation, Immunological disorders

## Abstract

Eosinophils are terminally differentiated cells derived from hematopoietic stem cells (HSCs) in the bone marrow. Several studies have confirmed the effective roles of eosinophils in asthmatic airway pathogenesis. However, their regulatory functions have not been well elucidated. Here, increased C-C chemokine ligand 6 (CCL6) in asthmatic mice and the human orthologs CCL15 and CCL23 that are highly expressed in asthma patients are described, which are mainly derived from eosinophils. Using *Ccl6* knockout mice, further studies revealed CCL6-dependent allergic airway inflammation and committed eosinophilia in the bone marrow following ovalbumin (OVA) challenge and identified a CCL6-CCR1 regulatory axis in hematopoietic stem cells (HSCs). Eosinophil differentiation and airway inflammation were remarkably decreased by the specific CCR1 antagonist BX471. Thus, the study identifies that the CCL6-CCR1 axis is involved in the crosstalk between eosinophils and HSCs during the development of allergic airway inflammation, which also reveals a potential therapeutic strategy for targeting G protein-coupled receptors (GPCRs) for future clinical treatment of asthma.

## Introduction

Asthma is among the most common chronic diseases, affecting 1–18% of the population in different countries with an increasing prevalence.^1^ A high blood eosinophil count is a predictive risk factor and biomarker of asthma exacerbations.^2,3^ Eosinophils differentiate from HSCs and mature in the bone marrow,^4^ are recruited to inflammatory sites, and release an array of cytokines, chemokines, or granules to mediate the airway pathological response, mucus hypersecretion, airway remodeling, and airway hyperresponsiveness.^5,6^ Directly targeting the eosinophil differentiation process is an effective therapeutic strategy to control clinical symptoms of asthma and reduce exacerbations.^7–11^

Eosinophils produce various cellular mediators involved in the occurrence and progression of allergic asthma. For example, active eosinophils produce cytokines, such as interleukin (IL)-4, IL-13, C-C chemokine ligand 5 (CCL5), and granulocyte-macrophage colony-stimulating factor (GM-CSF), and regulate dendritic cells and T helper type 2 (T_H_2) effector cells in pulmonary immune responses.^12–14^ Eosinophils also secrete granules, including eosinophil peroxidase (EPX), eosinophil granule major basic proteins, eosinophil cationic protein, and eosinophil-derived neurotoxin, which directly contribute to asthmatic pathology.^15,16^ These factors form an immunomodulatory network in allergic asthma, revealing the dynamic interplay between eosinophils and other immune cells.^17^ Recently, we found that eosinophils disrupt HSC homeostasis by impairing HSC maintenance and mobilization primarily through eosinophil-derived mCCL6.^18^ The regulatory effects of eosinophils on stem cells suggest that eosinophils may be involved in the initiation of asthmatic pathology. However, the complex roles of eosinophils and effective factors in allergic asthma are still not completely clear.

Reportedly, mCCL6 (also known as C10) is mainly produced by macrophages^19^ and attracts macrophages, CD4^+^ T cells, and eosinophils. mCCL6 plays important roles in inflammatory processes, including pulmonary fibrosis,^20^ allergic bronchopulmonary aspergillosis,^21^ sepsis,^22^ and experimental demyelinating diseases.^23^ Murine CCL6 shares homology with human CCL23 (also known as MPIF-1) and CCL15 (also known as MIP-5, MIP-1δ) and putatively activates CCR1.^24,25^ Our previous findings have shown the potential contribution of eosinophil-derived mCCL6 to HSC impairment in inflammatory airway disease.^18^ However, the precise function of mCCL6 is not well understood, and its pathogenic role in allergic asthma remains unknown; similarly, the roles of hCCL23 and hCCL15 in asthma patients remain to be explored.

In this study, we explored the functional role of eosinophil-derived mCCL6 in the pathology of allergic inflammation. mCCL6 interacts with CCR1, constituting a feedforward loop of asthma exacerbation. The human orthologs of mCCL6, hCCL23, and hCCL15, are also increased in asthma patients, supporting the clinical relevance of the current findings.

## Results

### Elevated hCCL23 and hCCL15 ortholog levels in asthma patients

**Fig. 1:**
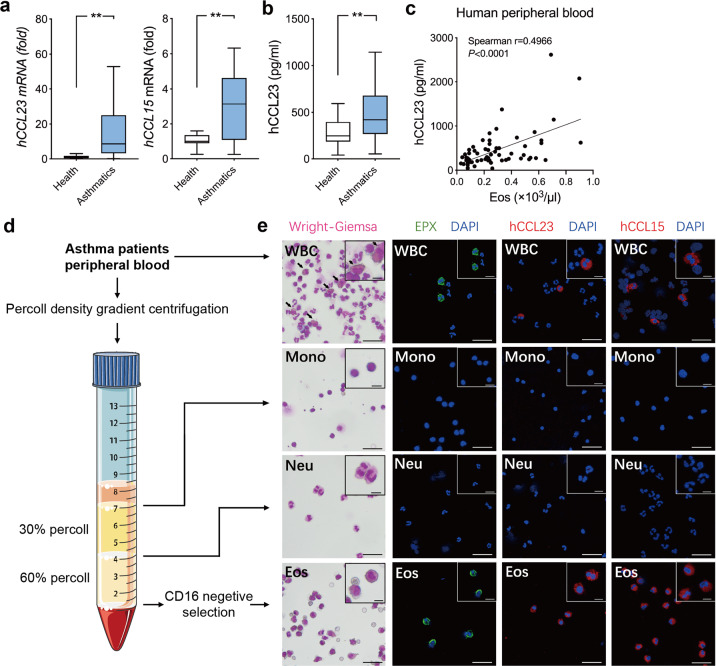
Increased expressions of hCCL23 and hCCL15 in asthma patients. **a** Relative mRNA expressions of *hCCL23* and *hCCL15* in total white blood cells (WBCs) from asthma patients (*n*=16) versus healthy control subjects (*n*=16). **b** hCCL23 concentration measured by ELISA in the plasma of asthma patients (*n*=31) compared with healthy control (*n*=30). Data in **a** and **b** are presented as median (centerline), and the whiskers indicate no more than 1.5 times the interquartile range (the difference between 25th and 75th percentiles, shown in the box). **c** Correlation of hCCL23 protein levels with the number of eosinophils in human peripheral blood (*n*=61, linear regression and Spearman rank correlation). **d** Schematic illustration of peripheral blood WBCs isolating procedure from asthma patients. **e** Representative images of total WBCs and isolated subsets slides of Wright-Giemsa staining and immunofluorescence staining for EPX (green), hCCL23 (red), hCCL15 (red), and DAPI (blue). Scale bar, 40μm. Insets show high-power images in the upper right corner of each overlay. Scale bar, 10μm. **P<0.01 by unpaired *t*-test

To explore the clinical relevance of hCCL23/hCCL15 and the eosinophil count in allergic asthma, we analyzed blood samples from 31 asthma patients with acute attack and 30 healthy controls. The clinical characteristics of the subjects are summarized in Table S1. We extracted the total mRNA from white blood cells (WBCs) and found that the relative mRNA expression levels of *hCCL23* and *hCCL15* in the asthma patients were higher than those in the controls (Fig. 1a). In addition, the serum hCCL23 concentrations in the asthma group were higher than those in the healthy control subjects (Fig. 1b) and correlated with the number of eosinophils in peripheral blood (Fig. 1c). Then, we separated human eosinophils, monocytes, and neutrophils in blood from asthma patients and performed immunofluorescence staining (Fig. 1d). Cells were verified through Wright-Giemsa staining and immunofluorescence staining. Notably, hCCL23 and hCCL15 expression was mainly found in eosinophils compared with other cell types in white blood cells (Fig. 1e and Supplementary Fig. S1a, b). Therefore, up-regulation of hCCL23 and hCCL15 in patients with asthma suggests a possible involvement of this cytokine in allergic airway inflammation.

### Eosinophil-derived mCCL6 is increased in a murine asthma model

**Fig. 2:**
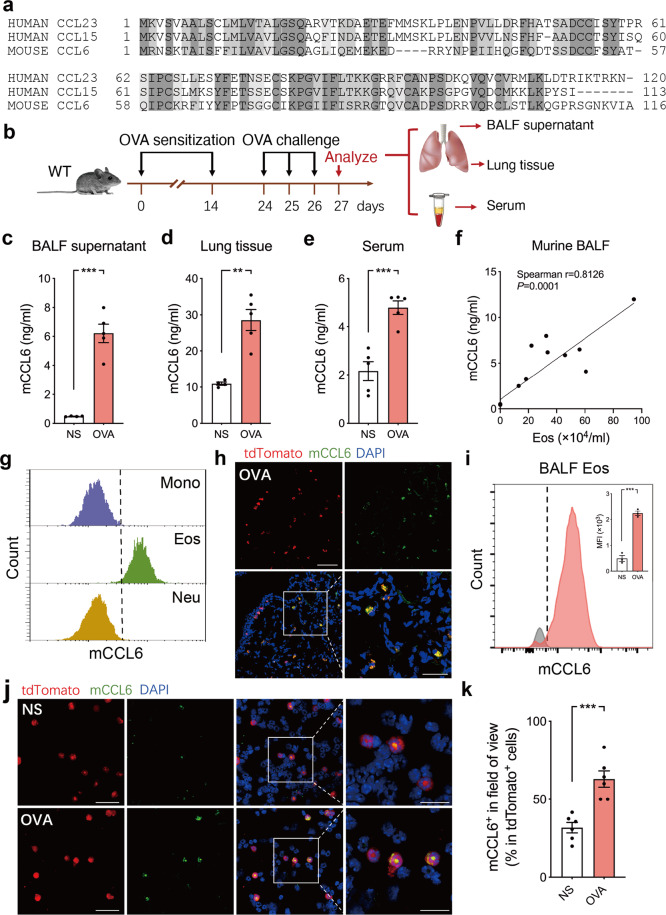
Increased expressions of eosinophil-derived mCCL6 in allergen-challenged mice. **a** Sequence alignment of the mCCL6 with the hCCL23 and hCCL15. *Shaded letters* indicate residues at each aligned position that are identical to the hCCL23 and hCCL15 specificity. *Dashes* indicate gaps that were inserted to optimize the alignment. **b** Schematic timeline and subsequent sample processing of allergic asthma mouse models. **c-e** Expressions of mCCL6 in BALF supernatant (**c**), lung tissue (**d**), and serum (**e**) measured by ELISA from NS or OVA-challenged mice. Each point represents an individual mouse; data from 4–5 mice per group are plotted as mean ± SEM. **f** Correlation of mCCL6 protein levels with the number of eosinophils in murine BALF (*n* = 17, some of the points are overlapped). **g** Representative histograms of mCCL6 expression in monocytes, eosinophils, and neutrophils from peripheral blood of NS mice. Cells were gated in Fig. S2a. **h** Representative lung immunofluorescence of eosinophils (tdTomato^+^, red) in OVA-challenged eoCre/R26-tdTomato mice staining with mCCL6 antibody (green) and DAPI (blue). Scale bar, 40 μm, 20 μm. **i** Representative histograms of mCCL6 expression in BALF eosinophils from OVA-challenged mice compared with NS mice. **j** Representative cytospin slides of bone marrow exhibited eosinophils (tdTomato^+^, red) in NS or OVA-challenged eoCre/R26-tdTomato mice staining with mCCL6 antibody (green) and DAPI (blue). Scale bar, 40 μm. **k** Quantitation of mCCL6^+^ percentage among tdTomato^+^ cells in **j** (*n* = 6 per group, 3 images per mouse). **P < 0.01; ***P < 0.001 by unpaired *t*-test

hCCL23 and hCCL15 are two of the four chemokines in the NC6 subfamily and are orthologs of mCCL6 (Fig. 2a). To study the roles of hCCL23 and hCCL15 in allergic airway inflammation, we established a murine model of ovalbumin (OVA)-induced allergic asthma and first examined the concentrations of the mCCL6 orthologs (Fig. 2b). We found that the mCCL6 levels in the bronchoalveolar lavage fluid (BALF) supernatant (Fig. 2c), lung tissues (Fig. 2d), and blood serum (Fig. 2e) from the OVA-challenged mice were all significantly higher than those from the control mice. Moreover, the mCCL6 levels were positively related to the eosinophil count in the BALF in a correlation analysis (Fig. 2f). To identify the sources of mCCL6, we analyzed mCCL6 in different sites of normal saline (NS) control and OVA mice. The intracellular mCCL6 levels in different cell types in peripheral blood were determined by flow cytometry (Supplementary Fig. S2a, b). The results showed that the mean fluorescence intensity (MFI) of mCCL6 in eosinophils was obviously higher than that in monocytes, neutrophils, and lymphocytes **(**Fig. 2g and Supplementary Fig. S2c), indicating that eosinophils were the main source of mCCL6 under homeostasis. Specific staining targeting EPX in BALF cells confirmed the co-localization of mCCL6 and EPX (Supplementary Fig. S2d). We further confirmed the source of mCCL6 by crossing eoCre mice with R26-tdTomato mice, which generated a mouse strain with specific tdTomato fluorescence for eosinophils. As expected, eosinophils were the predominant source of mCCL6 in inflammatory lung tissues (Fig. 2h and Supplementary Fig S2e). Furthermore, we analyzed the mCCL6 MFI in BALF eosinophils and found that compared with that in the control group (NS), the level of mCCL6 accumulation in eosinophils was more than twice that in the OVA group (Supplementary Fig. S2f and Fig. 2i). The immunofluorescence analyses of bone marrow cells showed that the eosinophil-derived mCCL6 level was elevated under OVA-induced airway inflammation (Fig. 2j, k), under which IL-5 might be a promoting factor of mCCL6 release through the MAPK pathway (Supplementary Fig. S2g). In an *Epx*-diphtherin transgenic mouse strain (Eos-null), mCCL6 was barely observed, along with attenuated airway inflammation in the asthmatic model (Supplementary Fig. S3). Taken together, these results reveal the predominant source of mCCL6 from eosinophils in both homeostasis and allergic airway inflammation and that the level of eosinophil-derived mCCL6 is increased in allergic conditions.

### CCL6-deficient mice exhibit alleviated eosinophilic airway inflammation

**Fig. 3:**
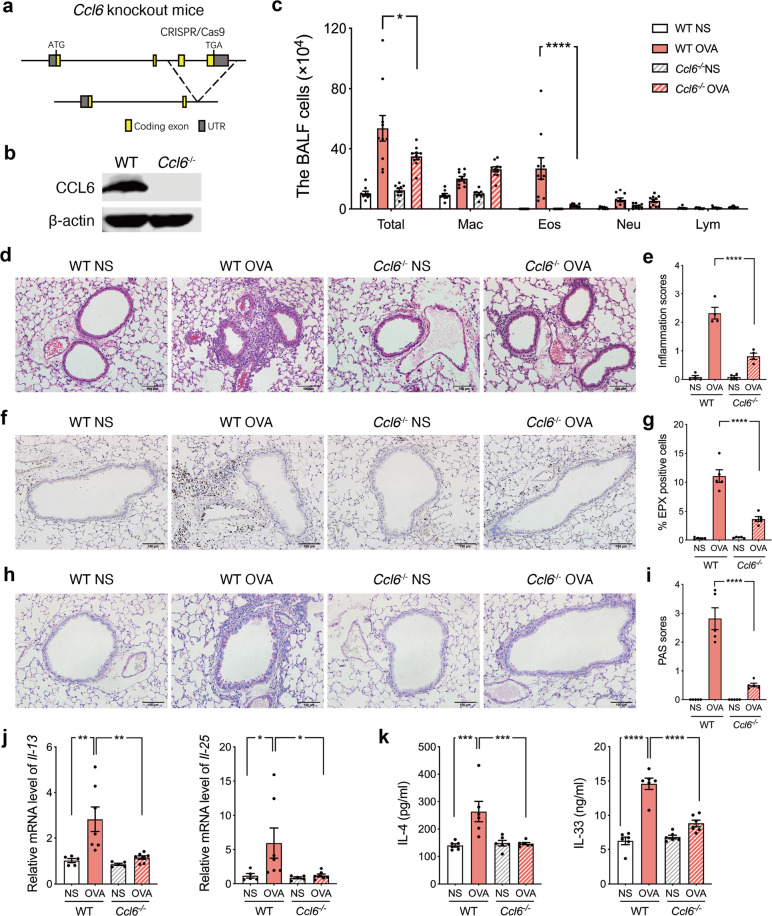
CCL6 deficiency alleviates OVA-induced eosinophilic airway inflammation. **a** Schematic map of established *Ccl6*^−/−^ mice by using the CRISPR/Cas9 system. **b** Representative blots of mCCL6 and β-actin (loading control) assessed by Western blot of protein extracts of eosinophils sorted from WT and *Ccl6*^−/−^ mice. **c** Differential counts on Wright-Giemsa stained BALF cells in WT and *Ccl6*^−/−^ mice. OVA-induced asthma model was presented in Fig. 1a. Combined data shown as mean ± SEM are presented for 9–10 mice per group from two independent experiments. Representative photomicrographs of lung sections with H&E staining (**d**), EPX staining (**f**), and those with PAS staining (**h**) at 24 h after the last OVA challenge. Scale bar, 100 μm. Histological inflammatory scores (**e**) and PAS scores (**i**) were analyzed from **d** and **h**. **g** The quantitative percentages of EPX^+^ cells in total nucleated cells analyzed from **f** (*n* = 4–5 mice per group, 4 images per mouse). **j** Relative mRNA levels of *Il-13* and *Il-25* in lung tissues were determined by quantitative RT-PCR at 24 h after the last NS or OVA challenge. **k** The concentration of IL-4 and IL-33 in lung tissue determined by ELISA. Data are mean ± SEM for 4–5 mice per group, 5–7 images per mouse. *P < 0.05; **P < 0.01; ***P < 0.001; ****, *P* < 0.0001 by one-way ANOVA with Sidak’s multiple comparisons test

To investigate the contribution of mCCL6 to asthma in vivo, we generated *Ccl6* knockout (*Ccl6*^*−/−*^) mice using the CRISPR/Cas9 system (Fig. 3a). The knockout efficiency was confirmed by Western blot analysis of mCCL6 from sorted eosinophils in the bone marrow of wild-type (WT) and *Ccl6*^*−/−*^ mice (Fig. 3b). Male and female *Ccl6*^*−/−*^ mice appeared healthy with no basal defects in complete blood cell counts (Supplementary Table S2). However, following the establishment of the OVA-induced asthma model, we found that the *Ccl6*^*−/−*^ mice exhibited a significantly decreased eosinophil count in BALF, whereas no significant effects were observed in the other cell types in BALF (Fig. 3c). Pathological lung section analysis showed that the OVA-challenged WT mice exhibited obvious inflammatory cell infiltration around the bronchi, while the *Ccl6*^*−/−*^ mice exhibited a significant attenuation of inflammatory infiltration (Fig. 3d, e), especially alleviation of eosinophilia, based on EPX staining (Fig. 3f, g). Periodic acid-Schiff (PAS) staining further revealed that the *Ccl6*^*−/−*^ mice displayed a mild response to the OVA challenge with less mucus secretion (Fig. 3h, i). Additionally, dual immunofluorescence staining of BALF cells with EPX and mCCL6 antibodies confirmed these results in OVA-challenged WT and *Ccl6*^*−/−*^ mice (Supplementary Fig. S4a).

Consistent with these findings, the increases in inflammation-related thymic stromal lymphopoietin (*Tslp*), *Epx* and secreted airway mucin *Muc5ac* mRNA expression observed in the lung tissues of the OVA-challenged WT mice were significantly attenuated in the *Ccl6*^*-/-*^ mice (Supplementary Fig. S4b). Using a T_H_2 cell flow cytometry gating strategy^26^ in the lung tissue (Fig. S5a), we found reduced infiltration of T_H_2 cells from lung tissues in the OVA-challenged *Ccl6*^-/-^ mice (Supplementary Fig. S5b, c) without an effect on the CD4^+^/CD8^+^ ratio (Supplementary Fig. S5d). We further evaluated typical T_H_2 cytokine production in lung tissues and found that upregulated mRNA expression of *Il-13* and *Il-25* (Fig. 3j) and elevated concentrations of IL-4 and IL-33 protein (Fig. 3k) in OVA-challenged WT mice were significantly attenuated in OVA-challenged *Ccl6*^*-/-*^ mice. These data indicate that OVA-induced airway inflammation is CCL6-dependent in vivo.

### CCL6-deficient mice display diminished eosinophilopoiesis under OVA challenge

**Fig. 4:**
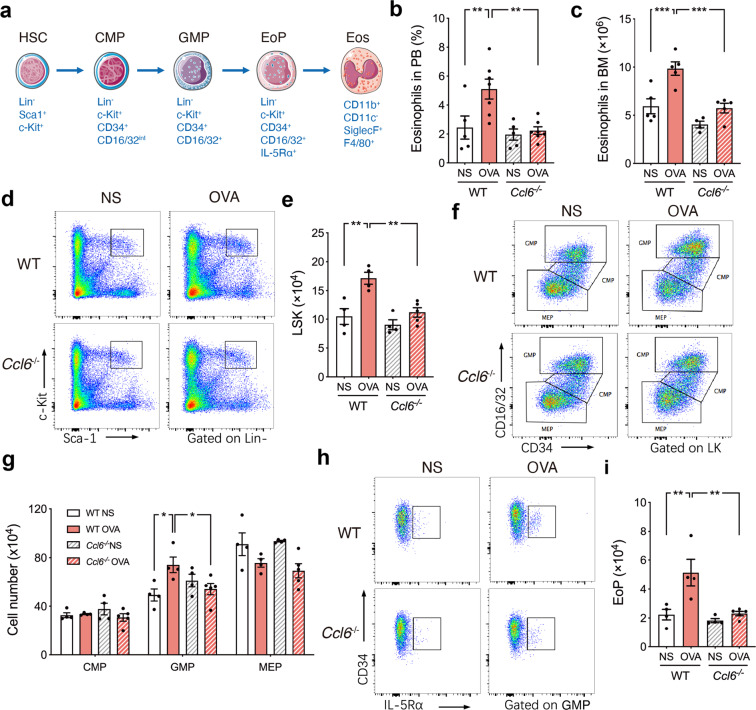
CCL6 deficiency abolishes the impairment of HSC homeostasis in allergen-induced airway inflammation. **a** Murine eosinophil differentiation hierarchy. **b-c** Quantitative number of eosinophils in peripheral blood (**b**) and bone marrow (**c**) of NS or OVA-challenged WT and *Ccl6*^-/-^ mice. **d-e** Representative flow cytometric dot plots (**d**) and quantitative number (**e**) of LSK. **f-g** Representative flow cytometric dot plots (**f**) and quantitative number (**g**) of CMP, GMP, MEP. **h**, **i** Representative flow cytometric dot plots (**h**) and quantitative number (**i**) of EoP. Data are statistically calculated as mean ± SEM for 4–6 mice in each group from three independent experiments. *P < 0.05; **P < 0.01; ***P < 0.001 by one-way ANOVA with Sidak’s multiple comparisons test

Our previous study identified that eosinophil-derived mCCL6 is an important mediator that activates HSCs under OVA-induced allergic airway inflammation. Several developmental stages of eosinophils from quiescent HSCs have been identified,^27^ including development of activated HSCs (lineage^−^Sca-1^+^c-Kit^+^, LSK), common myeloid progenitor cells (CMPs), and granulocyte-macrophage progenitor cells (GMPs) into eosinophil progenitor cells (EoPs) (Fig. 4a). The number of mature eosinophils in peripheral blood (Fig. 4b) and bone marrow (Fig. 4c) were not increased in OVA-challenged *Ccl6*^*-/-*^ mice, suggesting reduced eosinophil lineage differentiation with mCCL6 deficiency. First, we assessed the number of eosinophil-related progenitors in WT and *Ccl6*^*−/−*^ mice after OVA challenge by flow cytometry (Fig. S6). The proportion of HSCs identified as LSK was increased in the OVA-challenged WT mice but was only slightly changed in the *Ccl6*^*−/−*^ mice (Fig. 4d, e). Upon analyzing the lineages associated with eosinophilopoiesis, including CMPs, GMPs, megakaryocyte-erythroid progenitor cells (MEPs) (Fig. 4f, g), and EoPs (Fig. 4h, i), we found that mCCL6 deficiency abolished the increases in hematopoietic stem and progenitor cell populations in the bone marrow. These data demonstrate that mCCL6 is essential for eosinophil differentiation from HSCs in allergic airway inflammation.

### Identification of CCR1 as an mCCL6 receptor

**Fig. 5:**
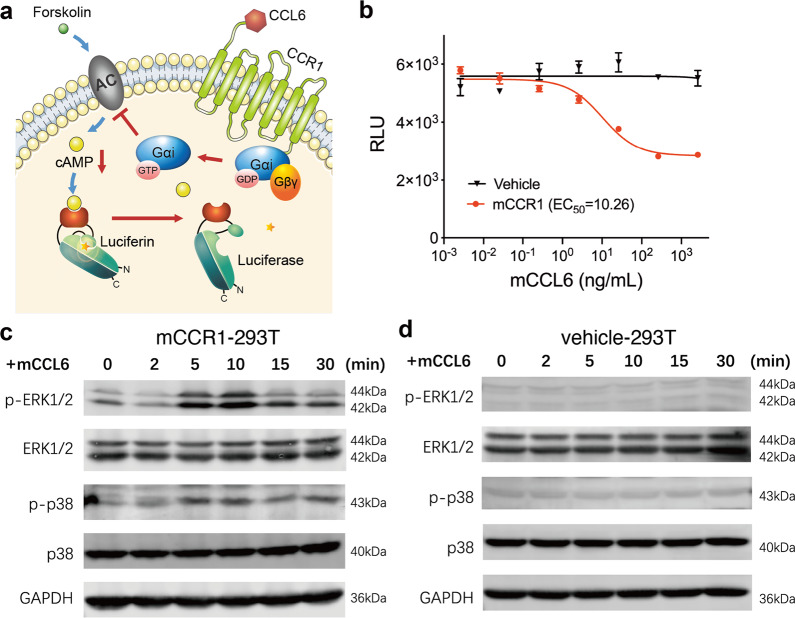
mCCL6 directly activates CCR1 and downstream signaling. **a** Schematic illustration of GloSensor assay in CCR1. Forskolin artificially elevated cAMP levels by activating adenylyl cyclase (AC) whereas CCR1 agonist inhibits AC activity. **b** Dose-response curves of the intracellular cAMP level measured by GloSensor assay. GloSensor-HEK293T cells transfected with vehicle or mCCR1 were treated with forskolin (1 μM) and indicated concentrations of mCCL6. The reduction of cAMP was recorded after 30 min. Data represent mean ± SEM of three technical replicates (error bars smaller than symbols are not shown). Median effective concentration (EC50) was calculated by nonlinear regression (three parameters). **c**, **d** Representative blots of p-ERK1/2, ERK1/2, p-p38, p38 and GAPDH (loading control) assessed by Western blot of protein extracts from mCCR1-293T (**c**) or vehicle-293T (**d**) cells cultured with 200 ng/ml mCCL6

The definite receptor of mCCL6 has not been precisely defined. A previous study reported that CCR1, which belongs to the G protein-coupled receptor (GPCR) superfamily, is a putative receptor of mCCL6 in IL-13-induced lung inflammation and remodeling models.^28^ Using a GloSensor assay (Fig. 5a), we first claimed that mCCL6 can activate mCCR1 to cause Gαi activation and downregulate the second messenger molecule cyclic AMP (cAMP) to trigger downstream signaling cascades (Fig. 5b, Supplementary Fig. S7a). Additionally, mCCL6 intervention in HEK293T cells transiently transfected with mCCR1 induced time-dependent p-ERK1/2 and p-p38 expression (Fig. 5c, d), providing evidence of mCCR1 activation. Similar to classical GPCRs,^29^ CCR1 is internalized following ligand binding and activation. Additionally, rapid intracellular calcium influx and later CCR1 internalization were found following CCL6 administration in primary eosinophils (Supplementary Fig. S7b, c).

### Specific CCR1 inhibition or depletion impairs committed eosinophil differentiation and attenuats airway inflammation

**Fig. 6:**
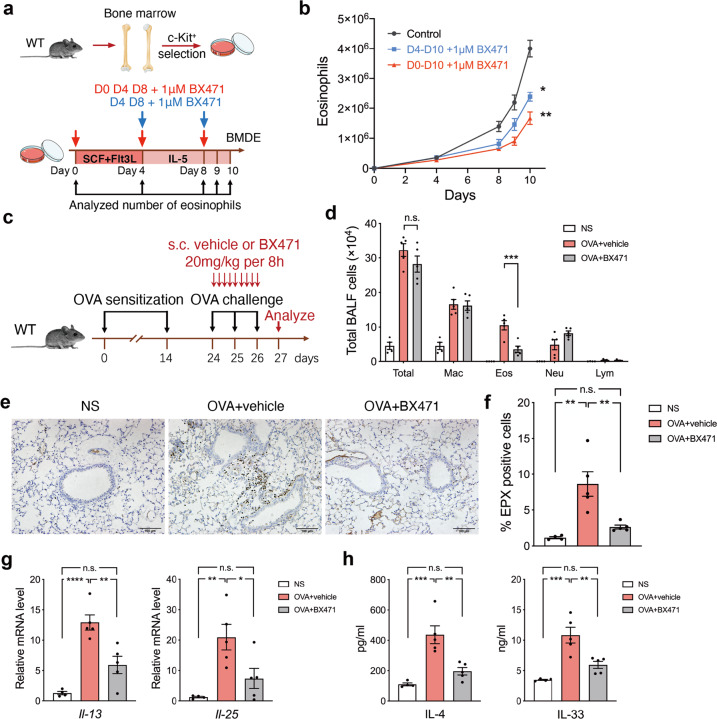
Inhibition of CCR1 alleviates OVA-induced lung eosinophilic inflammation. **a** Schematic of BMDE processing and culture timeline representing the treatment of BX471. **b** Number of eosinophils characterized as SiglecF^+^F4/80^+^ cells in WT BMDEs treated with BX471 as indicate in **a** (*n* = 4 mice per independent cultures). **c** Schematic timeline representing the treatment of BX471 in allergic asthma models. **d** Differential cell counts of cytospin preps represented as the number of BALF cells. **e** Representative images of lung tissues immunostained with EPX antibody after therapeutic treatment with BX471. Scale bar, 100 μm. **f** The quantitative percentages of EPX^+^ cells compared with total nucleated cells analyzed from **e** (*n* = 4–5 mice per group, 4 images per mouse). **g**, **h** Relative mRNA levels of *Il-13* and *Il-25* (**g**) and protein levels of IL-4 and IL-33 (**h**) in lung tissues determined by quantitative RT-PCR and ELISA. Data are mean ± SEM for 4–5 mice in each group from two independent experiments in **d**, **f**–**h**. n.s., not significant; *P < 0.05; **P < 0.01 ***P < 0.001; ****P < 0.0001 by two-way ANOVA in **b**, one-way ANOVA with Tukey’s *post hoc* test in **d**, **f**–**h**

We hypothesized that the interaction between mCCL6 and CCR1 contributes to eosinophilia and allergic inflammation. Next, we generated bone marrow-derived eosinophils (BMDEs) and treated them with BX471, which is a potent, specific CCR1 antagonist (Fig. 6a). BX471 administration on days 0, 4, and 8 or days 4 and 8 resulted in diminished eosinophil development on days 8, 9, and 10 (Fig. 6b). Similar results were confirmed in the differentiation of BMDEs from *Ccr1* knockout (*Ccr1*^*-/-*^) mice (Supplementary Fig. S8), suggesting that CCR1 inhibition can decrease committed eosinophil differentiation.

Next, we analyzed the expression and activation of CCR1 in sorted Lin^−^ cells from the bone marrow in asthmatic models. Although no difference in CCR1 expression was found in NS and OVA mice, the cell surface CCR1 level on lineage- cells was significantly decreased in the OVA group, representing ligand recognition and CCR1 internalization (Supplementary Fig. S7a, b). We then established an OVA-challenged asthma model with conditional CCR1 depletion in the hematopoietic system by total bone marrow transplantion to analyze eosinophil differentiation (Supplementary Fig. S10a). CCR1-deficient bone marrow cells exerted attenuated eosinophilic lineage differentiation (Supplementary Fig. S10b–e). Furthermore, a chimeric model with WT and *Ccr1*^*-/-*^ bone marrow cells was generated, in which stem cells and progenitors shared the same microenvironment under differentiation stress caused by OVA challenge-induced mCCL6 stimulation (Supplementary Fig. S11a). There were less committed eosinophil differentiation from *Ccr1*^*−/−*^ stem cells or progenitors, including GMPs and EoPs, and even mature eosinophil than from the WT cells (Supplementary Fig. S11b–d).

Correspondingly, we further explored the effects of specific CCR1 inhibition on allergic airway inflammation in vivo. Mice were subcutaneously injected with BX471 (20 mg/kg) or vehicle every 8 h during the period of NS or the OVA challenge (Fig. 6c). In BALF cells, OVA-induced eosinophilia was fully reversed by BX471 treatment (Fig. 6d). EPX^+^ eosinophil infiltration around the bronchi was decreased after BX471 treatment of lung tissue to levels comparable to those in the NS-challenged mice (Fig. 6e, f). Analysis of mCCL6 levels showed that BX471 treatment significantly decreased mCCL6 in the lung tissue and BALF (Supplementary Fig. S12a−c), suggesting that mCCL6 levels were decreased with the change of eosinophils. Analysis of the relative mRNA levels of *Il-13* and *Il-25* (Fig. 6g) and the protein levels of IL-4 and IL-33 (Fig. 6h) in the lung tissues also showed that the increases in representative T_H_2 cytokines were successfully abolished in the mice treated with BX471. These data suggest that the blockade of mCCL6-CCR1 signaling prevents eosinophilic inflammation in the lungs.

## Discussion

In this study, we found increases in hCCL23 and hCCL15 in asthma patients. The animal studies showed similar results consisting of increased expression of eosinophil-derived mCCL6, which was responsible for eosinophilic airway inflammation. Using *Ccl6*^*-/-*^ mice, we found that a CCL6 deficiency significantly decreased OVA-induced eosinophilia, mucus hypersecretion, and T_H_2 responses in the airways. Specifically, we provide direct evidence that mCCL6 activates CCR1 and induces downstream p-ERK1/2 and p-p38 expression. Treatment with the specific CCR1 antagonist BX471 significantly relieved eosinophil differentiation and OVA-induced eosinophilic airway inflammation both in vitro and in vivo. Thus, we concluded that the CCL6-CCR1 axis is an important regulatory mechanism in asthma pathogenesis and a potential target for further therapeutic research.

Our previous and current studies emphasize the functions of a critical chemokine, i.e., mCCL6, which is mainly derived from eosinophils, that promotes committed eosinophil development and initiates allergic airway inflammation by directly acting on bone marrow HSCs. Eosinophils are multifunctional leukocytes, and evidence supports the broader roles of the interactions between eosinophils and progenitor cells, which may act as potent effector cells, in the initiation and promotion of allergic inflammation.^30^ The blockade of progenitors at the source of HSCs or inhibition of eosinophil differentiation in the bone marrow has been implicated as an effective therapeutic strategy for asthma in animal studies.^31,32^ Here, we identified mCCL6 as an essential mediator involved in the crosstalk between eosinophils and progenitor cells in a mouse model of allergic inflammation. Increases in HSC and EoP populations critically depend on the upregulation of mCCL6 expression, which likely participates in the setting of allergic inflammation, suggesting that mCCL6 contributes to a potential feedback effect between eosinophils and their progenitor cells in pulmonary pathologies.

HSCs are responsible for the lifelong production of blood cells. Furthermore, HSCs must respond to acute or chronic needs, such as injury or inflammation. It has long been known that proinflammatory cytokines, such as IL-1, tumor necrosis factor-α (TNF-α), and interferons (IFNs), can promote HSC proliferation and increase the cellular output of bone marrow cells.^33^ In this regulatory cycle of chronic inflammation, we note that a high level of mCCL6 leads to unbalanced HSC differentiation compared to that in the homeostatic state. Thus, we hypothesize that the continuous production of mCCL6 from eosinophils is a trigger of HSC dysfunction and the chronic inflammation regulation cycle. Our study provides new insight into how chronic inflammatory signals affect HSCs to sustain the airway pathological response.

Chemokines are small proteins that function as immune modulators through the activation of GPCRs to mediate immune cell trafficking. Chemokines and their receptors have emerged as prominent players and key therapeutic targets in a wide range of immune and inflammatory disorders.^34^ However, the biology and biochemistry of inflammatory chemokines and chemokine receptors are complex partially because the receptors display promiscuous ligand binding, and in turn, the chemokines bind multiple chemokine receptors. mCCL6, hCCL15, and hCCL23 belong to the NC6 subfamily with N-terminal extensions.^25^ hCCL23, which binds the cell surface receptor CCR1, has been reported to be associated with total IgE in children with asthma.^35^

Previous studies have shown that hCCL15 binds the cell surface receptor CCR1 and may contribute to the severity of asthma and airflow limitation by affecting airway smooth muscle cells.^36^ Furthermore, we provide evidence that mCCL6 activates CCR1 downstream of the Gαi protein and related phosphorylated signaling proteins to activate HSCs and promote HSC differentiation, providing convincing evidence of the CCL6-CCR1 axis. A number of CCR1 antagonists have recently been identified and studied in the context of inflammatory diseases, and these antagonists have shown potential therapeutic effectiveness in clinical trials.^37^ These data suggest that targeting the CCL6-CCR1 axis could be a promising therapeutic strategy for preventing or ameliorating allergic inflammation.

In summary, this study shows that the levels of the hCCL23 and hCCL15 orthologs are elevated in asthma patients and demonstrates the essential role of eosinophil-derived mCCL6 in a murine allergic asthma model and allergen-induced eosinophilia. Airway inflammation is mediated by mCCL6 and diminished in mice with mCCL6 deficiency or following specific CCR1 inhibition. Thus, a better understanding of the new functional role of the CCL6-CCR1 interaction in eosinophil differentiation during allergic airway inflammation may lead to novel therapeutic targets for the development of allergic asthma treatments. Our findings also contribute to a new theory of chemokine-receptor function as biomarkers and suggest that targeting NC6 family chemokines and related receptors with effective neutralizing antibodies or specific inhibitors might be potential therapeutic strategies for eosinophilic airway inflammation.

## Materials and methods

### Human samples

Thirty-one asthma patients and 30 healthy volunteers were recruited from the Second Affiliated Hospital of Zhejiang University School of Medicine. All subjects were informed and signed informed consent prior to inclusion in the study. The clinical characteristics of the subjects are present in Table S1. Cohort inclusion criteria for all subjects were: age between 18 and 70 years old and able to complete this study and all tests. For the patients with asthma, inclusion criteria were: patients with acute attack who meet the diagnosis of asthma. For the non-asthmatic controls, the following criteria were essential for inclusion: the absent history of asthma, no use of asthma-related medication, no pulmonary obstruction, and no upper or lower respiratory tract infection or related symptoms. All human studies were approved by the Ethics Committee for Human Studies of Second Affiliated Hospital of Zhejiang University School of Medicine (2019 NO.460).

### Mice

C57BL/6 mice were purchased from Shanghai SLAC Laboratory Animal Co., Ltd. (Shanghai, China). *Ccl6* knockout (*Ccl6*^*-/-*^) mice were generated by the Nanjing Biomedical Research Institute of Nanjing University. *Il-5* transgenic mice (*Il-5* Tg), *Epx*-Cre (eoCre) mice were a gift from the late Dr. J. J. Lee (Mayo Clinic). *Rosa26-loxp-STOP-loxp-tdTomato* mice (R26-tdTomato) were kindly offered by Dr. C. Liu (Zhejiang University). All the mice were on C57BL/6 background and maintained under specific-pathogen-free conditions in the Laboratory Animal Center of Zhejiang University. The genotypes of mice were confirmed by PCR analysis. All animal experimental protocols were approved by the Ethics Committee for Animal Studies of Second Affiliated Hospital of Zhejiang University School of Medicine (2019-452).

### Murine asthma model, BX471 treatment, and assessments of airway inflammation

Mice aged 8–10 weeks were used for establishing asthma models shown in Fig. 2b as described previously.^38^ Briefly, on days 0 and 14, mice were sensitized by an intraperitoneal injection of 20 μg OVA (Sigma-Aldrich) emulsified in 2.25 mg Imject™ Alum (77161, Thermo Scientific) in a total volume of 200 μL. On days 24–26, sensitized mice were subsequently challenged with an aerosol generated from 1.5% OVA in NS by an ultrasonic atomizer (NE-C900, OMRON). Mice were killed 24 h after the last challenge for data acquisition.

BX471 hydrochloride (HY-12080A, MCE) treatment was given subcutaneously on days 24–26 (per 8 h) at a dose of 20 mg/kg^39^ as shown in Fig. 6c. For control mice, all treatments were replaced with NS or vehicle.

Inflammatory cells in BALF from left lungs were collected for inflammation evaluation. BALF cells were then counted by using a light microscope and centrifuged with a cytospin (Cytospin 4, Thermo Scientific) followed by Wright-Giemsa staining according to the manufacturer’s instructions to identify the cell type. Lung sections from formalin-fixed/paraffin-embedded tissue were additionally stained to assess inflammation and mucus accumulation. Hematoxylin and eosin (H&E) stained and periodic acid-Schiff (PAS) stained sections were assigned a score on an arbitrary scale of 0–4 as previously described for the inflammatory situation.^40^ Lung tissue eosinophils were evaluated by staining with mouse antibody against eosinophil-specific EPX (5 μg/mL, Mayo Clinic). Photomicrographs of lung sections were obtained by an Olympus BX53 microscopy and cellSens Dimension software (Olympus). The EPX^+^ cells and total nucleated cells were calculated by ImageJ v1.52 software from at least 4 images per mouse.

### Percoll density gradient separation of human WBCs

Eosinophils, neutrophils, and monocytes from healthy controls and asthma patients were isolated by a density gradient separation. Briefly, a 60% and 30% (v/v) Percoll/synthetic medium mixture (GE Health care) was prepared and carefully placed in a 15 mL sterile centrifuge tube at equal volume (3 mL). Blood samples mixed with PBS (1:1 v/v, total 3 mL) were then carefully placed onto the upper layer of 30% Percoll. Tubes were centrifuged at 800 × *g* (acceleration 4, deceleration 0) for 30 min at room temperature. The mononuclear cell fraction (plasma/30% Percoll interphase), granulocyte fraction (30%/60% interphase), and eosinophil fraction (on top of RBCs) were isolated, and all fractions were treated Lysing buffer (555899, BD Biosciences) and washed in 50 mL PBS by centrifugation at 400 × *g* for 5 min. To further purify eosinophils, cells from eosinophil fraction were incubated with human CD16 MicroBeads (Miltenyi Biotec). Eosinophils were then enriched by negative selection and neutrophils were enriched by positive selection using a magnetized MACS Column (Miltenyi Biotec) and identified by Wright-Giemsa staining and EPX immunofluorescence staining.

### mRNA extraction and quantitative RT-PCR

Total mRNA in lung tissues from mice or RBCs-removed peripheral blood cells from human samples were isolated with a TRIzol Plus reagent (Takara) and used for the cDNA synthesis with the PrimeScript™ RT reagent Kit (Takara). Quantification of target gene transcripts was done by quantitative polymerase chain reaction using TB Green^®^ Premix Ex Taq™ (Tli RNaseH Plus) (Takara) on a StepOne Plus Real-Time PCR system (Applied Biosystems), with the expression of *β-actin* as internal control. The sequences of primers used are listed in Table S3.

### ELISA

Lung tissues were weighed and homogenized in RIPA lysis buffer containing 10 mM PMSF and a protease inhibitor cocktail (1 g/12 μL). Homogenates were ultrasonicated and centrifuged at 12,000 rpm for 10 min at 4 °C, and the supernatants were collected. mCCL6 concentrations in the serum (1:5 dilution), lung tissue (1:100 dilution) and BALF supernatant (1:10 dilution) were detected by a Mouse CCL6/C-C Motif Chemokine 6 ELISA Kit (RAB0557, Sigma-Aldrich). IL4 and IL33 concentrations in lung tissues were detected by an IL-4 Mouse ELISA Kit (BMS613, Invitrogen) and an IL-33 Mouse ELISA Kit (1:20 dilution, BMS6025, Invitrogen), respectively. CCL23 concentrations in the serum of human samples were detected using a Human MPIF-1 ELISA Kit (RAB0065, Sigma-Aldrich). All these above-mentioned detections were performed according to the relevant manufacturer’s instructions.

### Flow cytometry

A volume of 20 μL peripheral blood and total BALF cells was collected from NS and OVA-challenged mice. After lysis of RBCs, cells were stained with the following antibodies: anti-CD45 (clone 30-F11, eBioscience), anti-SiglecF (clone E50-2440, BD Biosciences), anti-Gr-1 (clone RB6-8C5, BioLegend), anti-CX3CR1 (clone SA011F11, BioLegend), anti-CD125 (clone T21, BD Biosciences), anti-CD11b (clone M1/70, eBioscience), and Fixable Viability Stain (FVS, BD Biosciences) was used to exclude dead cells. For mCCL6 staining, cells were first incubated with anti-CD16/CD32 (clone 2.4G2, BD Biosciences) to avoid nonspecific binding. After surface antibodies staining, BALF cells were fixed and permeabilized beforehand with Fixation/Permeabilization buffers (BioLegend) then stained with mouse CCL6/C10 antibody (clone 262016, R&D systems). Cells were gated as described in Fig. S2a, b and f. MFI was used to evaluate the expression of mCCL6 in these cells.

For T_H_2 cells in the lung, lungs were perfused with PBS via the right ventricle, and lung lobes were cut into small pieces then digested in a final volume of 2 mL RPMI-1640 (Gibco) containing 1 mg/mL collagenase I (Sigma-Aldrich) and 100 μg/mL DNase I (Sigma-Aldrich) at 37 °C for 45 min. Digested lung tissues were gently ground and filtered to obtain single-cell suspensions, and blocked with anti-CD16/CD32 antibodies. The staining strategy in Fig. S5a, as described previously.^26^ Cells were counted with a cell counter (CounterStar) and then stained with anti-CD3 (clone 17A2, BioLegend), anti-CD4 (clone GK1.5, BD Biosciences), anti-CD8a (clone 53-6.7, eBioscience), anti-CD278/ICOS (clone C398.4 A, BioLegend), anti-IL-33R/ST2 (clone RMST2-2, Invitrogen).

For eosinophils, progenitors, and stem cells staining in the bone marrow, both femurs and tibias from individual mice were collected and flushed with cold PBS, then crushed in PBS containing 0.5% BSA using a mortar and pestle for collecting single-cell suspensions. Cells were counted with a cell counter and adjusted to 1 × 10^8^ cells/mL, from which 20 μL and 100 μL were respectively taken for eosinophil and progenitor analysis. For eosinophils staining, cells were stained with anti-CD45, anti-SiglecF, and anti-F4/80 (clone BM8, BioLegend) antibodies. For LSK/progenitors staining, cells were first stained with biotin-conjugated anti-mouse lineage cocktail antibodies including Ter-119 (clone TER-119), Gr-1 (clone RB6-8C5), CD11b (clone M1/70), CD45R/B220 (clone RA3-6B2), CD4 (clone RM4-5) and CD8 (clone 53–6.7) (all from BioLegend) for labeling lineage-negative (Lin^−^) cells and then stained with the following antibodies: Streptavidin (BioLegend), anti-c-Kit (clone ACK2, eBioscience), anti-Sca-1 (clone El3-161.7, BioLegend), anti-CD34 (clone RAM34, BD Biosciences), anti-CD16/32 (clone 93, BD Biosciences) and anti-CD125/IL-5Rα. For CCR1 staining, cells were further stained with anti-CCR1 (clone S15040E, BioLegend) or Rat IgG2b kappa Isotype Control (clone eB149/10H5, eBioscience). All staining reactions were performed at 4 °C for 30 min, protected from light. DAPI (1 μg/mL, D9542, Sigma) was used to exclude dead cells. The gating strategies are described in Fig. S6. All the above data were acquired using a BD LSRFortessa (BD Biosciences) or a CytoFlex analyzer (Beckman Coulter). Data were analyzed using FlowJo™ 10 or CytExpert 2.0 software.

### Immunofluorescence staining

After lysis of RBCs, separated human WBCs and BALF cells or bone marrow cells from mice were centrifuged onto microscope slides on a cytospin (Thermo Scientific) for subsequent intracellular staining. Mouse lung tissues were first perfused with 50% optimal cutting temperature (OCT) compound and 10% sucrose in PBS, then frozen in OCT before cutting to 10 μm slices. The slides were then fixed in 4% paraformaldehyde for 10 min, and permeabilized with 0.5% Triton X-100 (Sigma-Aldric h) in PBS containing 5% FBS for 20 min, blocked with 5% goat serum (Sangon) in PBS for 30 min. The primary antibodies incubated for 4 °C overnight are listed as follows: mouse anti-human CCL23 (1:50 dilution, sc-393897, Santa Cruz), mouse anti-MIF-5 (hCCL15, 1:50 dilution, sc-398069, Santa Cruz), mouse anti-EPX (1:500 dilution, Mayo Clinic), rabbit anti-C10 (1:1000 dilution, ab191400, Abcam), rat anti-MBP (1:50 dilution, Mayo Clinic), rabbit anti-CD3 zeta (1:500 dilution, ab40804, Abcam). Secondary antibodies applied for 40 min at room temperature are conjugated of AF488 anti-mouse IgG, AF555 anti-mouse IgG, AF488 anti-rabbit IgG, AF555 anti-rabbit IgG, AF488 anti-rat (all from Invitrogen) at 1:2000 dilution. After counterstained with 1 μg/mL DAPI, the slides were mounted with a Fluoromount-G™ (SouthernBiotech). *Z*-stack images were acquired on an Olympus IX83-FV3000-OSR confocal microscopy and processed with the FV31S-SW software (Olympus).

### Murine eosinophils isolation and Western blot

To obtain large numbers of eosinophils, WT and *Ccl6*^*−/−*^ mice were intraperitoneally injected with 1 mL 3% Fluid Thioglycollate medium (Millipore) for 3 days consecutively. On day 4, mice were sacrificed to collect peritoneal-derived cells for sorting SiglecF^+^F4/80^+^ eosinophils.

Eosinophils and HEK293T were lysed in RIPA lysis buffer (biosharp) containing 10 mM PMSF, cOmplete™ Protease Inhibitor Cocktail, and PhosSTOP™ (all from Roche), then ultrasonicated and centrifuged at 12,000 rpm for 10 min at 4 °C. Subsequently, protein concentrations in supernatants were quantified by a Pierce BCA Protein Assay Kit (23225, Thermo Scientific). Equivalent amounts of protein from each sample were resolved through 15% SDS-PAGE (Epizyme Biotech), then transferred to PVDF membranes (Millipore), and immunoblotted with antibodies against rabbit anti-C10 (1:500 dilution), mouse anti-β-Actin (1:2000 dilution, Santa Cruz), rabbit anti-Phospho-p42/44 MAPK (ERK1/2) (clone D13.14.4E, 1:2000 dilution, Cell Signaling Technology), mouse anti-p42/44 MAPK (ERK1/2) (clone 3A7, 1:1000 dilution, Cell Signaling Technology), mouse anti-Phospho-p38 MAPK (clone 28B10, 1:2000 dilution, Cell Signaling Technology), rabbit anti-p38 MAPK (clone D13E1, 1:1000 dilution, Cell Signaling Technology), and rabbit anti-GAPDH (clone EPR16891, 1:10000 dilution, Abcam). After incubation with DyLight 680 or DyLight 800 goat anti-rabbit IgG (H + L) and DyLight 680 or DyLight 800 goat anti-mouse IgG (H + L) (all for 1:2000 dilution, EarthOx), the membranes were scanned using Odyssey CLx and quantified using Image Studio v5.2.5 (LI-COR Biosciences) software.

### GloSensor cAMP assay

Measurement of recombinant mouse CCL6/C10 truncated (aa 42–116, R&D systems)-induced cAMP down-regulation in HEK293T cells transiently expressing mouse CCR1 was performed using the GloSensor cAMP biosensor (Promega) according to the manufacturer’s protocols. The schematic illustration is shown in Fig. 5a. Briefly, HEK293T cells were placed into a 6-well plate at 2.5 × 10^5^/mL overnight and then transfected with both 500 ng pGloSensor-22F cAMP plasmid and 1500 ng mouse CCR1 plasmid or pcDNA3.1 vehicle plasmid per well. Twenty-two hours after transfection, cells were then placed into poly-D-lysine-coated 96-well plates (10^5^/well). Another 24 h later, cell medium were replaced by 50 μL of CO_2_-independent medium with 2% GloSensor cAMP Reagent (E1291, Promega) and incubated for 2 h at 37 °C. Then cells were stimulated with mCCL6 at a concentration ranging from 3.3 × 10^3^ to 3.3 × 10^−3^ ng/mL, followed by 1 μM forskolin (MCE) treatment. After 30 min, the relative light unit (RLU) of averaged firefly luciferase activity was measured on a Cytation 3 reader (BioTek) and presented as dose-response curves. Results are confirmed by three independent experiments.

### Bone marrow-derived Eosinophils (BMDE) differentiation and BX471 treatment

Bone marrow eosinophil cultures were prepared as described previously^41^ with slight modification. Bone marrow cells were harvested from femurs and tibias of WT and *Ccl6*^-/-^ mice. After removing RBCs, c-Kit^+^ hematopoietic progenitor cells were enriched by positive selection with APC-conjugated c-Kit antibody and anti-APC MicroBeads (Miltenyi Biotec). Cells were resuspended to a density of 1 × 10^6^/mL in Iscove’s Modified Dulbecco’s Medium (Gibco) supplemented with 10% FBS (Invitrogen), 2 mM L-glutamine (GlutaMAX™, Gibco), 5 × 10^−5^ M β-ME (Sigma-Aldrich), MEM Non-Essential Amino Acids (11140050, Gibco), 1 mM sodium pyruvate (11360070, Gibco), and 100 U/mL penicillin/streptomycin. The cytokines murine SCF (100 ng/mL, PeproTech) and murine Flt3-Ligand (100 ng/mL, PeproTech) were supplemented to the culture for 4 days. On day 4 and day 8, cells were washed and treated in the presence of IL-5 (10 ng/mL, R&D systems) for the duration of the culture. For BX471 treatment, 1 μM BX471 was added on days 0, 4, and 8 (blue arrow in Fig. 6a), or days 4 and 8 (red arrow in Fig. 6a). Flow cytometry analyses of eosinophils were performed on days 4 and 8–10. The number of total cells was counted with a cell counter.

### Statistical analysis

For comparison between two groups, data were analyzed by two-tailed unpaired *t*-test. When more than two groups were compared, data were analyzed by one-way or two-way ANOVA with the Sidak correction (when compared to some of the group) or Tukey *post hoc* test (when compared to every group) for multiple comparisons. The correlations were assessed by using linear regression and Spearman rank correlation. All statistical analyses were performed with Prism 8 for macOS (GraphPad Software) and considered statistically significant when *P* values < 0.05.

## Supplementary information

Supplementary Materials

## Data Availability

All data associated with this study are available in the main text or the supplementary materials.
