# Supplementary material for: Eosinophil-derived chemokine (hCCL15/23, mCCL6) interacts with CCR1 to promote eosinophilic airway inflammation
Source: Signal Transduct Target Ther. 2021 Feb 28;6:91. doi: 10.1038/s41392-021-00482-x (PMC7914252; doi:10.1038/s41392-021-00482-x)
Supplement: Supplementary file 1 — Supplementary Materials [file 41392_2021_482_MOESM1_ESM.docx]

Supplementary Materials for

Eosinophil-derived Chemokine (hCCL15/23, mCCL6) Interacts with CCR1 to Promote Eosinophilic Airway Inflammation

Xufei Du, Fei Li, Chao Zhang, Na Li, Huaqiong Huang, Zhehua Shao, Min Zhang, Xueqin Zhan, Yicheng He, Zhenyu Ju, Wen Li, Zhihua Chen, Songmin Ying^*^ and Huahao Shen^*^

Correspondence to: huahaoshen@zju.edu.cn and yings@zju.edu.cn

**This file includes:**

Materials and Methods

Figures. S1 to S12

Tables S1 to S3

**Materials and Methods**

***Mice***

*Cd3δ-Il-5* transgenic mice (*Il-5* Tg), which have expansion of eosinophil population, and *Epx*-diphtherin transgenic (Eos-null) mice were gifts from the late Dr. J. J. Lee (Mayo Clinic). *Ccr1* knockout (*Ccr1^-/-^*) mice were generated by Cyagen (Cyagen Biosciences Inc., China). All the mice were on C57BL/6 background and maintained under specific-pathogen-free conditions in the Laboratory Animal Center of Zhejiang University. The genotypes of mice were confirmed by PCR analysis. All animal experimental protocols were approved by the Ethics Committee for Animal Studies of Second Affiliated Hospital of Zhejiang University School of Medicine (2019-452).

***Eosinophils purification***

Eosinophils were obtained and purified from peripheral blood of *Il-5* Tg or *Il-5* Tg/*Ccl6^-/-^*mice. Leucocytes were isolated by Percoll density gradient separation laid on a four-layer gradient of 80%, 60%, 55% and 45% Percoll. Cells contained eosinophils from the 60%/80% interface were harvest and washed twice in PBS. Eosinophils were subsequently isolated through removing the contaminating lymphocytes and other granulocytes by negative selection with biotin antibodies for CD45R (B220), CD4, CD8, NK1.1, Ly-6G, CD115, CD11c and Ter-119 (all from BioLegend) and biotin conjugated magnetic beads. The purity of the eosinophils was determined by visual examination of Wright-Giemsa's staining and flow cytometry analysis. Then eosinophils were used for cell culture, intracellular calcium analysis and performing internalization assay.

***Intracellular calcium analysis***

Fluo-4-acetoxymethyl ester (AM) Ca^2+^ probe (Solarbio, CN) was used for measuring the intracellular Ca^2+^ concentration. In brief, fresh isolated eosinophils were incubated in Fluo-4 AM working solution (4 μM in HBSS with 1% FBS) at 37 °C in a 5 % CO2-containing incubator for 30 min. Then cells were washed with HBSS thrice to fully remove the AM esters and suspended with DPBS. Cells were separated in two groups treated with mCCL6 (400ng/mL) or vehicle. The fluorescence intensity of intracellular Ca^2+^ in each group was assayed via flow cytometry.

***Internalization assay***

Eosinophils were treated for 2 h with vehicle control or mCCL6 to evaluate mCCR1 cell-surface expression and ligand-induced internalization. Cells were washed twice and stained with mCCR1 antibody (BioLegend), then analyzed on CytoFlex (Beckman Coulter).

**Fig. S1**

**
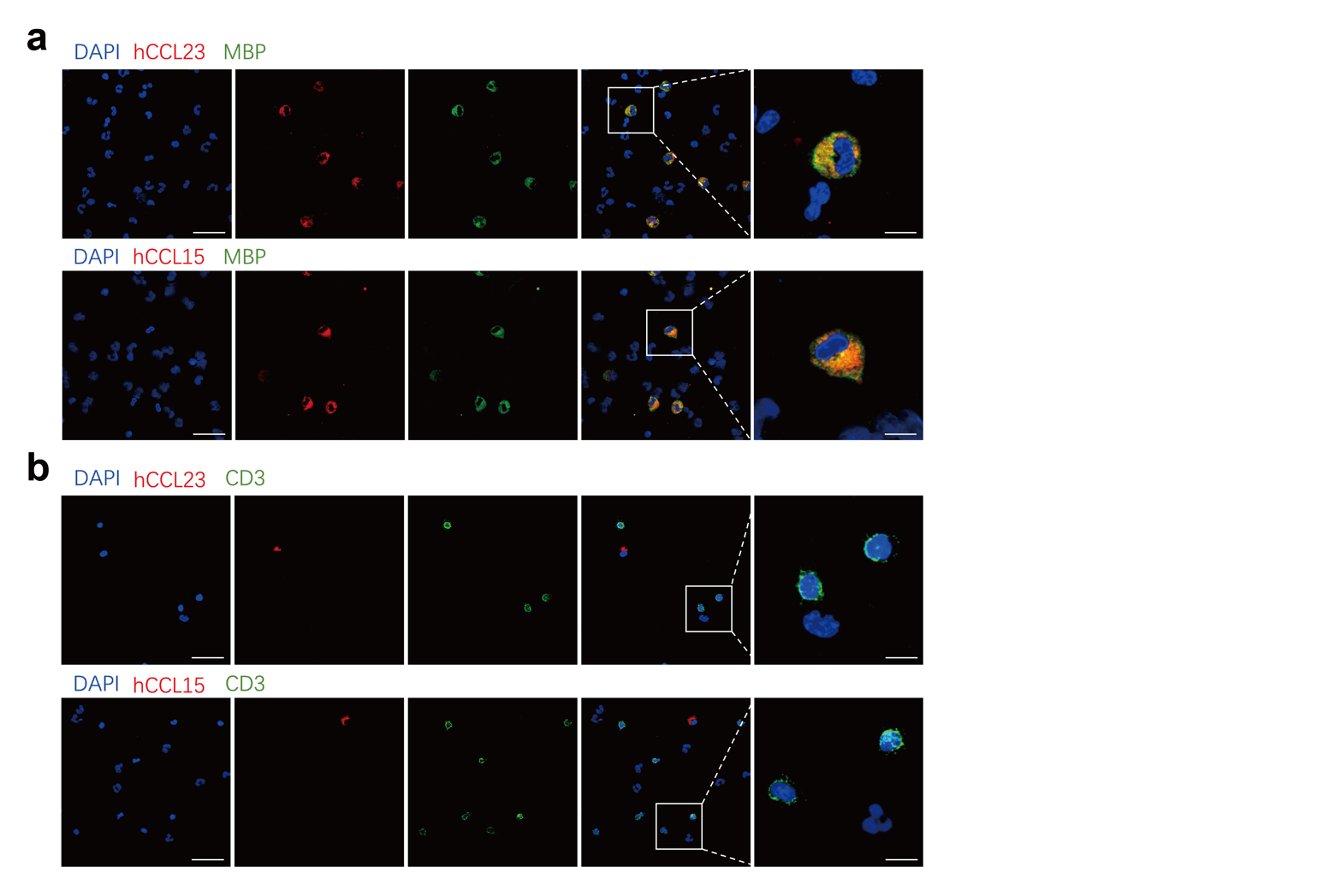
**

**Fig. S1 No expression of hCCL23 and hCCL15 in human white blood cells except eosinophils.**

**a** Expression of hCCL23 and hCCL15 by eosinophils in peripheral blood cells by dual immunofluorescence staining with antibodies against hCCL23/hCCL15 (red), MBP (green) followed by confocal microscopy after labelling with DAPI (blue). **b** T lymphocytes are not found to express hCCL23 and hCCL15. CD3 (green) and hCCL23/hCCL15 (red) followed by confocal microscopy after labelling with DAPI (blue). Scale bar, 40μm. A zoom three magnified image of cells highlighted in the overlay is shown (far right panel). Scale bar, 10μm.

**Fig. S2**


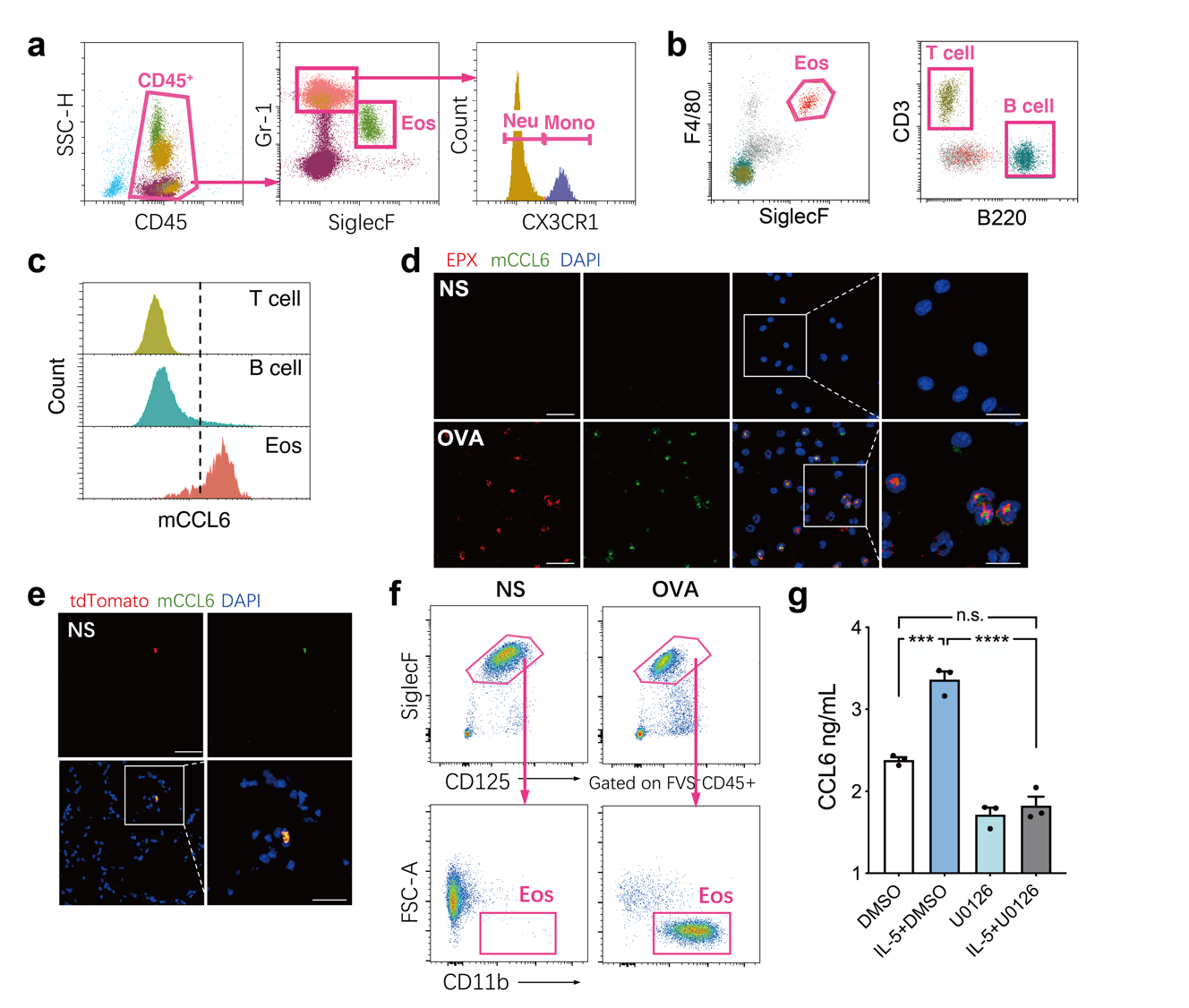


**Fig. S2** **Murine eosinophils are the main source of mCCL6 in allergic inflammation.**

**a** Gating strategy of WBCs in murine peripheral blood. Eosinophils (Eos) were defined as CD45^+^SiglecF^+^Gr-1^int^, monocytes (Mono) were CD45^+^Gr-1^hi^SiglecF^-^CX3CR1^+^, and neutrophils (Neu) were CD45^+^Gr1^hi^SiglecF^-^CX3CR1^-^. **b** Gating strategy of T cells (CD45^+^CD3^+^) and B cells (CD45^+^B220^+^) in murine peripheral blood. **c** Representative histograms of mCCL6 expression in T cells, B cells compared to Eos from peripheral blood of NS mice. Cells were gated in **b**. **d** Representative images of Eos (EPX^+^, red) in BALF after NS or OVA-challenged staining with mCCL6 antibody (green) and DAPI (blue). Scale bar, 40 μm, 20 μm. **e** Representative lung immunofluorescence by Eos (tdTomato+, red) in basal status of lungs from eoCre/R26-tdTomato mice staining with mCCL6 antibody (green) and DAPI (blue). Scale bar, 40 μm, 20μm. **f** Flow cytometry gating strategy for determination of Eos in murine BALF cells. **g** ELISA assay to detect the expression of mCCL6 in Eos (3×10^6^/ml) under recombinant IL-5 (20ng/ml) with or without U0126 (20μM) treatment for 6h.

**Fig. S3**

**
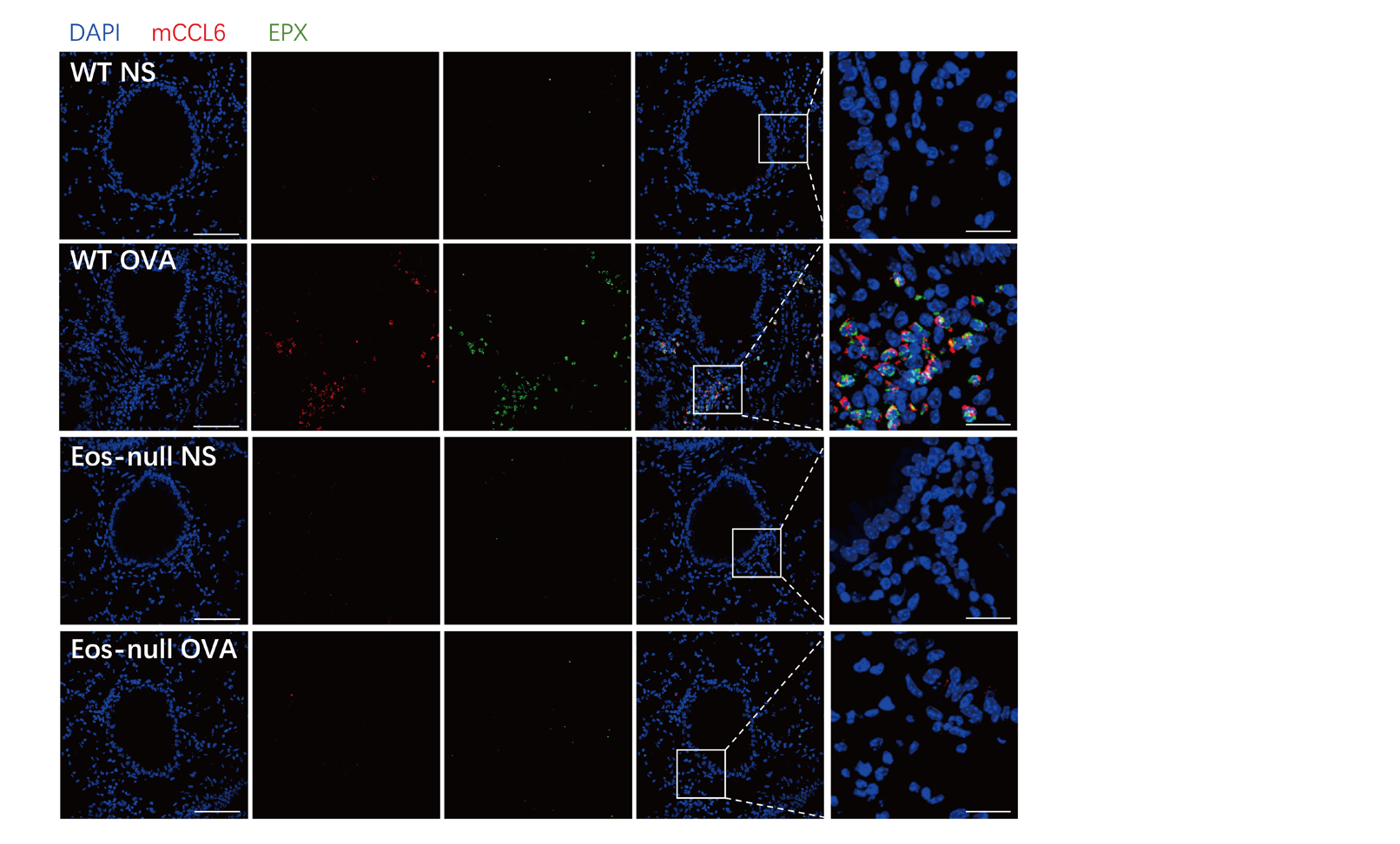
**

**Fig. S3** **mCCL6 expression is** **barely observed in allergic inflammatory models of Eos-null mice.**

Representative images of Eos (EPX^+^, green) in lung tissue of WT and Eos-null mice after NS or OVA-challenged staining with mCCL6 antibody (red) and DAPI (blue). Scale bar, 40 μm. A zoom three magnified image of cells highlighted in the overlay is shown (far right panel). Scale bar, 20 μm.

**Fig. S4**

**
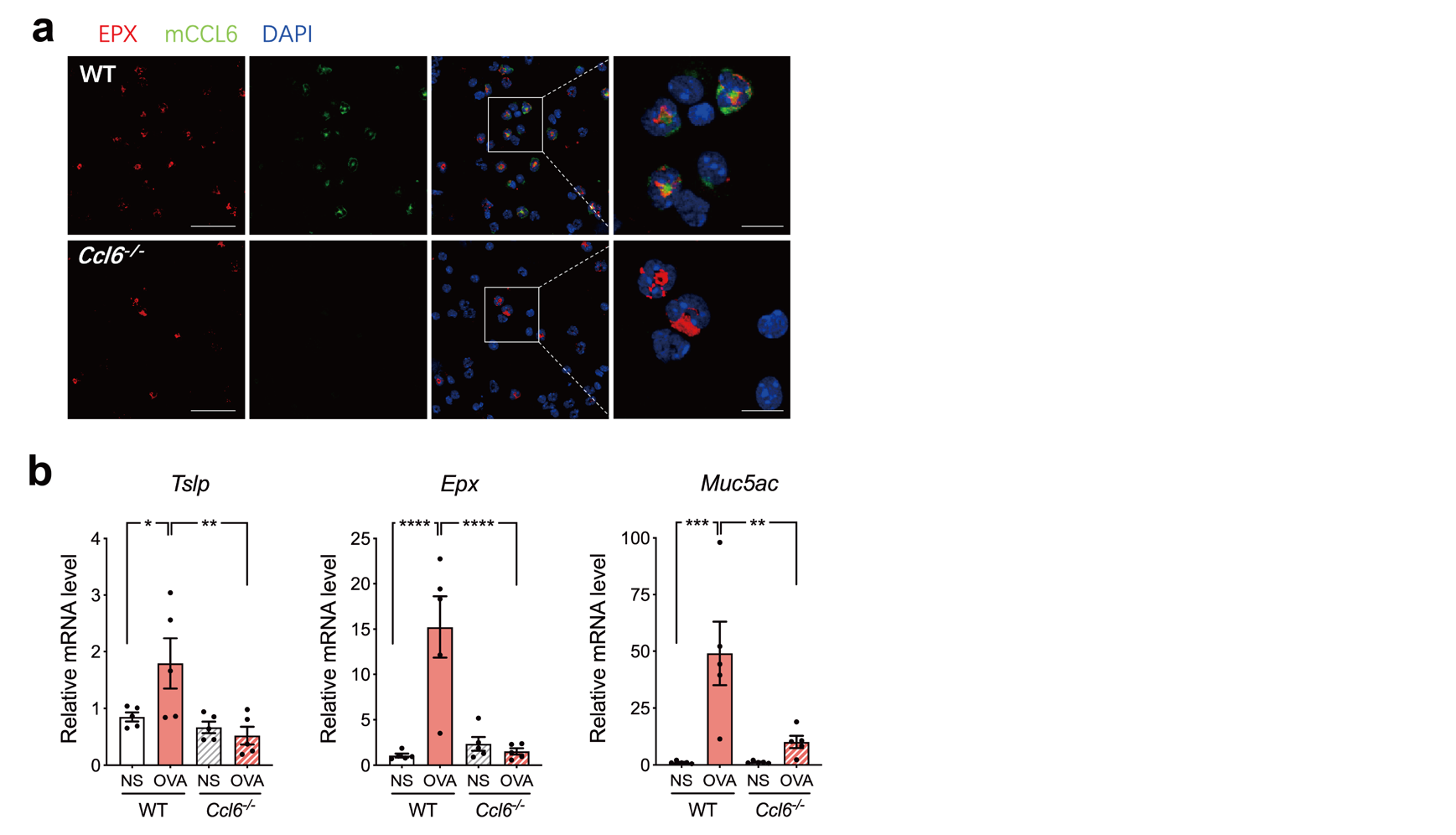
**

**Fig. S4 Immunofluorescence of BALF cells and quantitative RT-PCR of lung tissue in OVA-challenged WT and *Ccl6^-/-^* mice.**

**a** Representative image of BALF cells stained with EPX (red), mCCL6 (green) and DAPI (blue) followed by confocal microscopy. Scale bar, 40 μm. Right panels show high-power images of the outlined areas. Scale bar, 20 μm. **b** Relative mRNA level of *Tslp*, *Epx* and *Muc5ac* in lung tissues were determined by quantitative RT-PCR at 24 hours after last NS or OVA challenge. *, *P* < 0.05; **, *P* < 0.01; ***, *P* < 0.001; ****, *P* < 0.0001 by one-way ANOVA with Sidak’s multiple comparisons test.

**Fig. S5**


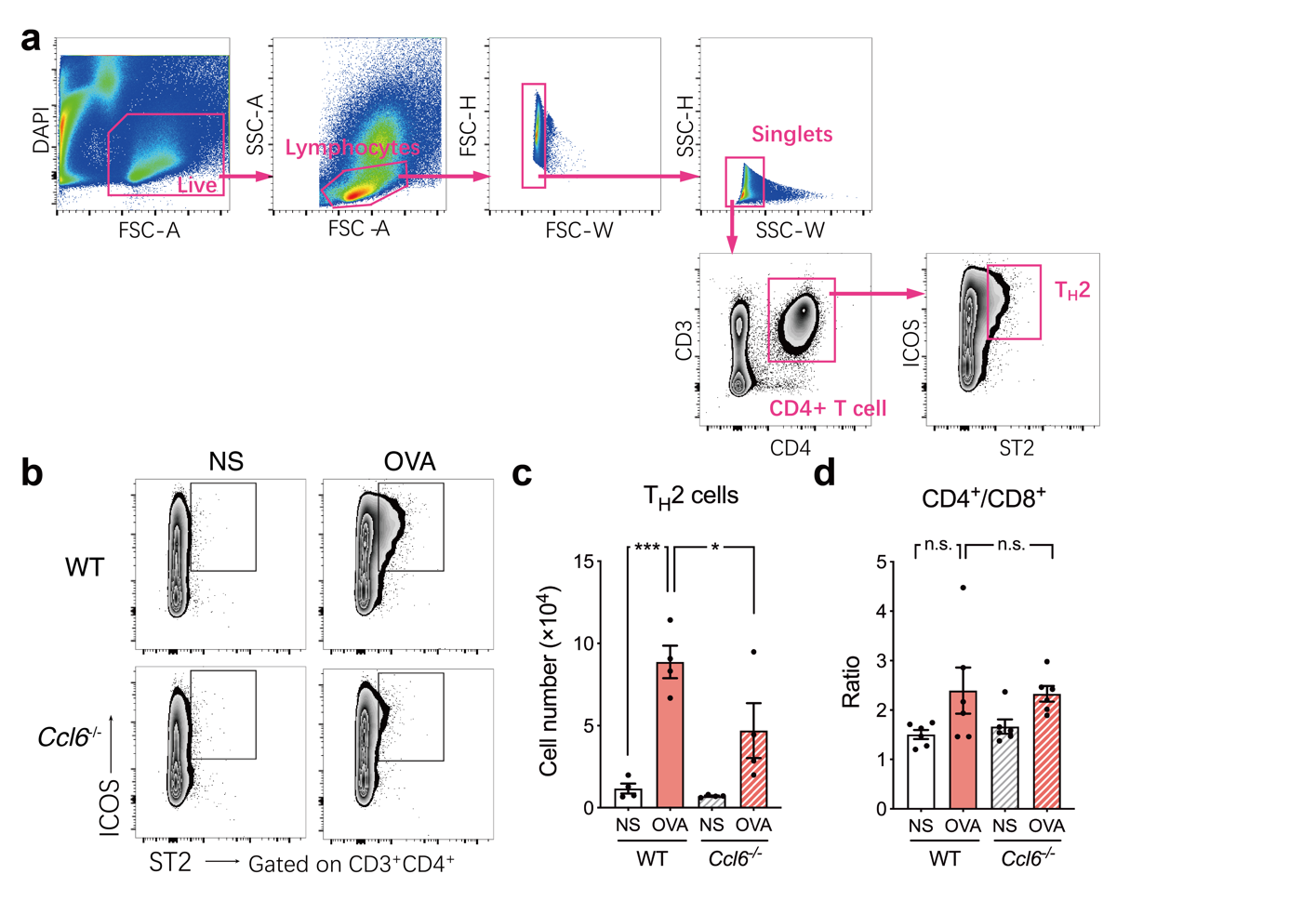


**Fig. S5**. **mCCL6 deficiency impaired T_H_2 cells infiltration.**

**a** Gating strategy for determination of T_H_2 cells in lung tissue. **b** Representative flow cytometric dot plots of T_H_2 cells characterized as CD3^+^CD4^+^ICOS^+^ST2^+^ in the lung tissue from WT and *Ccl6^-/-^* mice challenged with NS or OVA. **c** Quantification of T_H_2 cell numbers in **b**. **d** The ratio of CD4^+^ to CD8^+^ T cells. Data are mean ± SEM for 4-6 mice in each group from three independent experiments. n.s., not significant; *, *P* < 0.05; ***, *P* < 0.001 by one-way ANOVA with Sidak’s multiple comparisons test.

**Fig. S6**


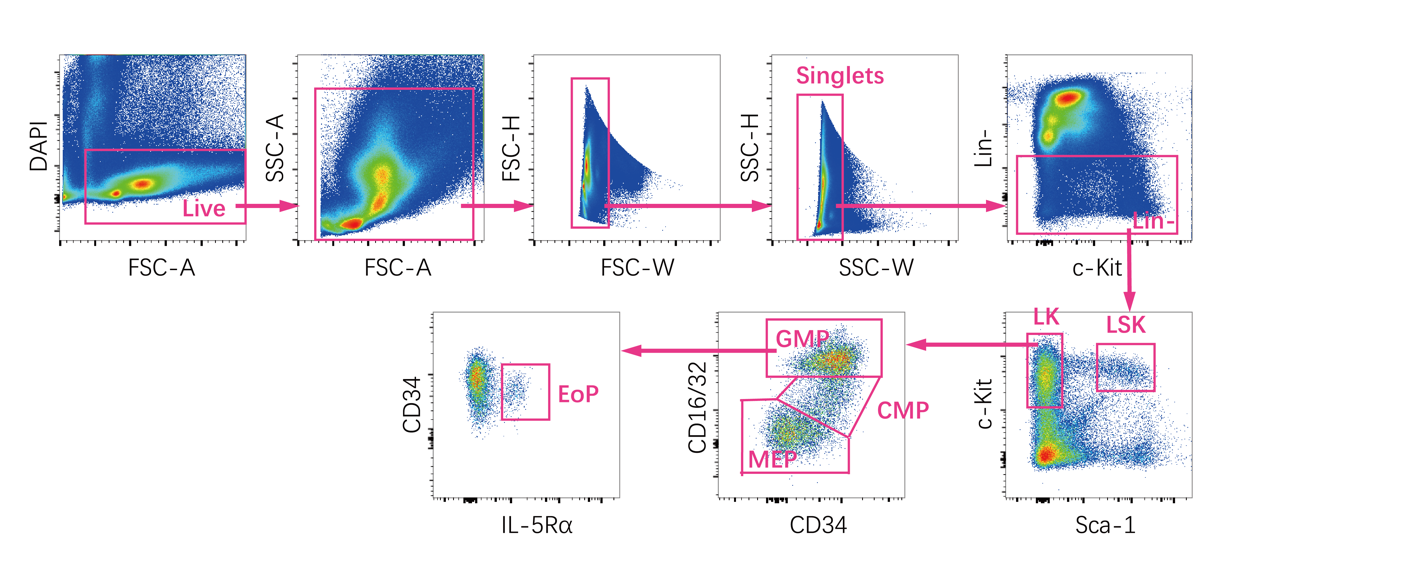


**Fig. S6**. **Flow cytometry gating strategy of hematopoietic stem and progenitor cells.**

Gating strategy for determination of hematopoietic stem and progenitor cells in bone marrow.

**Fig. S7**

**
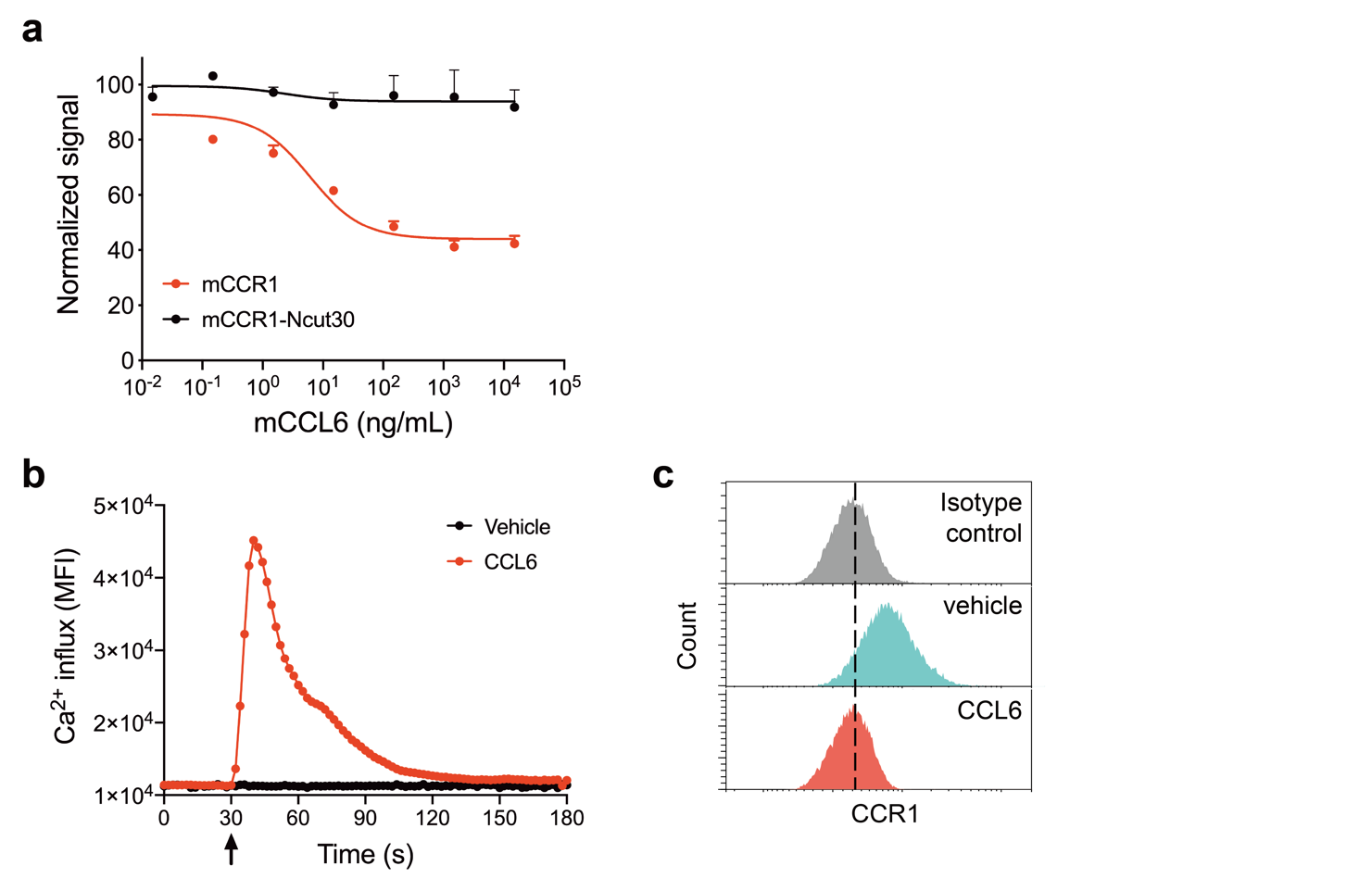
**

**Fig. S7 mCCL6 binds and activates CCR1.**

**a** Dose-response curves of normalized intracellular cAMP signal measured by GloSensor assay. GloSensor-HEK293T cells transfected with mCCR1 or mCCR1-Ncut30 were treated with forskolin (1 μM) and indicated concentrations of mCCL6. The reduction of cAMP was recorded after 30 min. Data represent mean ± SEM of three technical replicates (error bars smaller than symbols are not shown). **b** Quickly intracellular calcium was detected in primary eosinophils (deletion of mCCL6) treated with vehicle or mCCL6 (400ng/mL) at 30 s (arrow indicate) then record for another 150 s by flow cytometry. **c** mCCR1 surface expression was examined in primary eosinophils (deletion of mCCL6) treated with vehicle or mCCL6 (400ng/mL) for two hours. Results were analyzed by flow cytometry.

**Fig. S8**


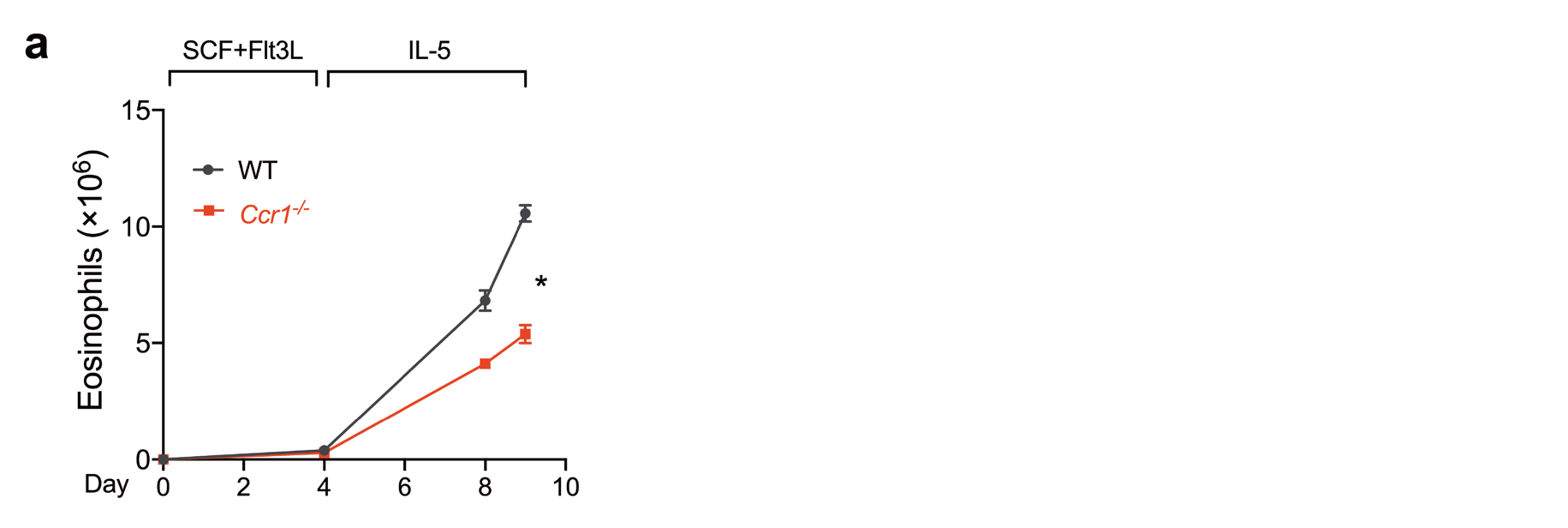


**Fig. S8 CCR1 deficiency decreases eosinophil differentiation *in vitro*.**

Numbers of eosinophils characterized as SiglecF^+^F4/80^+^ cells in BMDEs from WT and *Ccr1^-/-^* mice (n=3 mice per independent cultures). *, *P* < 0.05 by two-way ANOVA.

**Fig. S9**


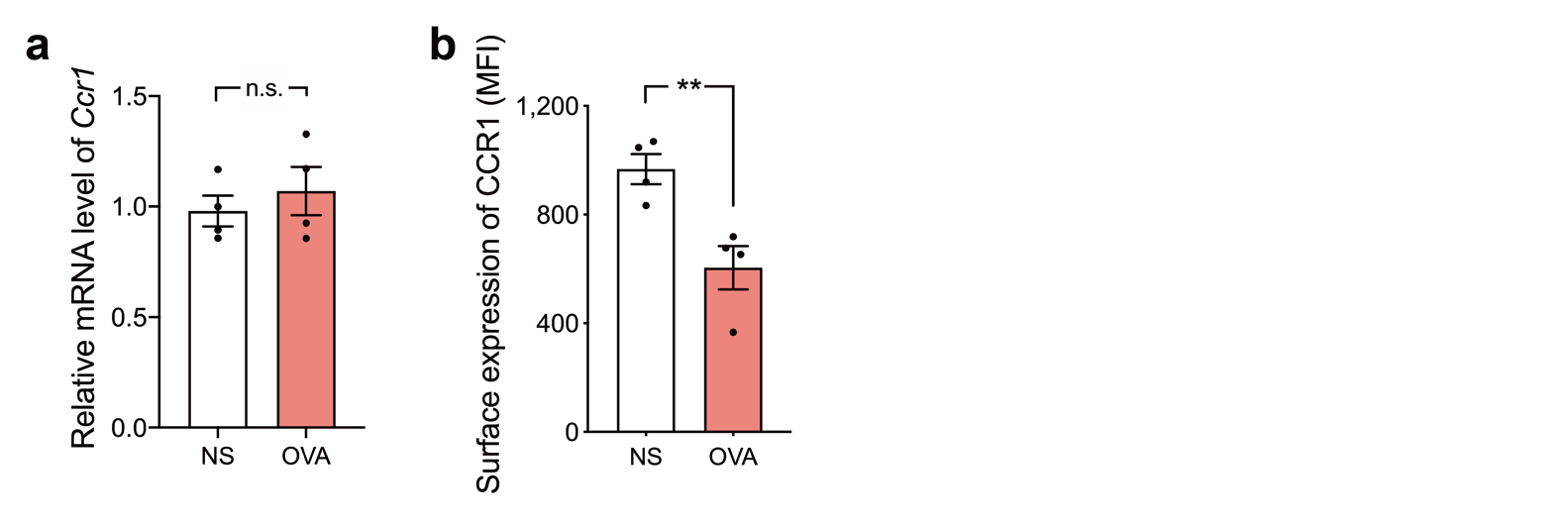


**Fig. S9**. **CCR1 expression and activation in Lin- cells from bone marrow of allergic inflammatory model.**

**a** Quantitative RT-PCR analysis of CCR1 mRNA expression in sorted lineage^-^ cells in bone marrow from NS and OVA mice. **b**. mCCR1 surface expression were examined on lineage- cells in NS and OVA mice. Data are statistically calculated as mean ± SEM for 4 mice per group. n.s., not significant; **, *P* < 0.01 by unpaired *t* test.

**Fig. S10**


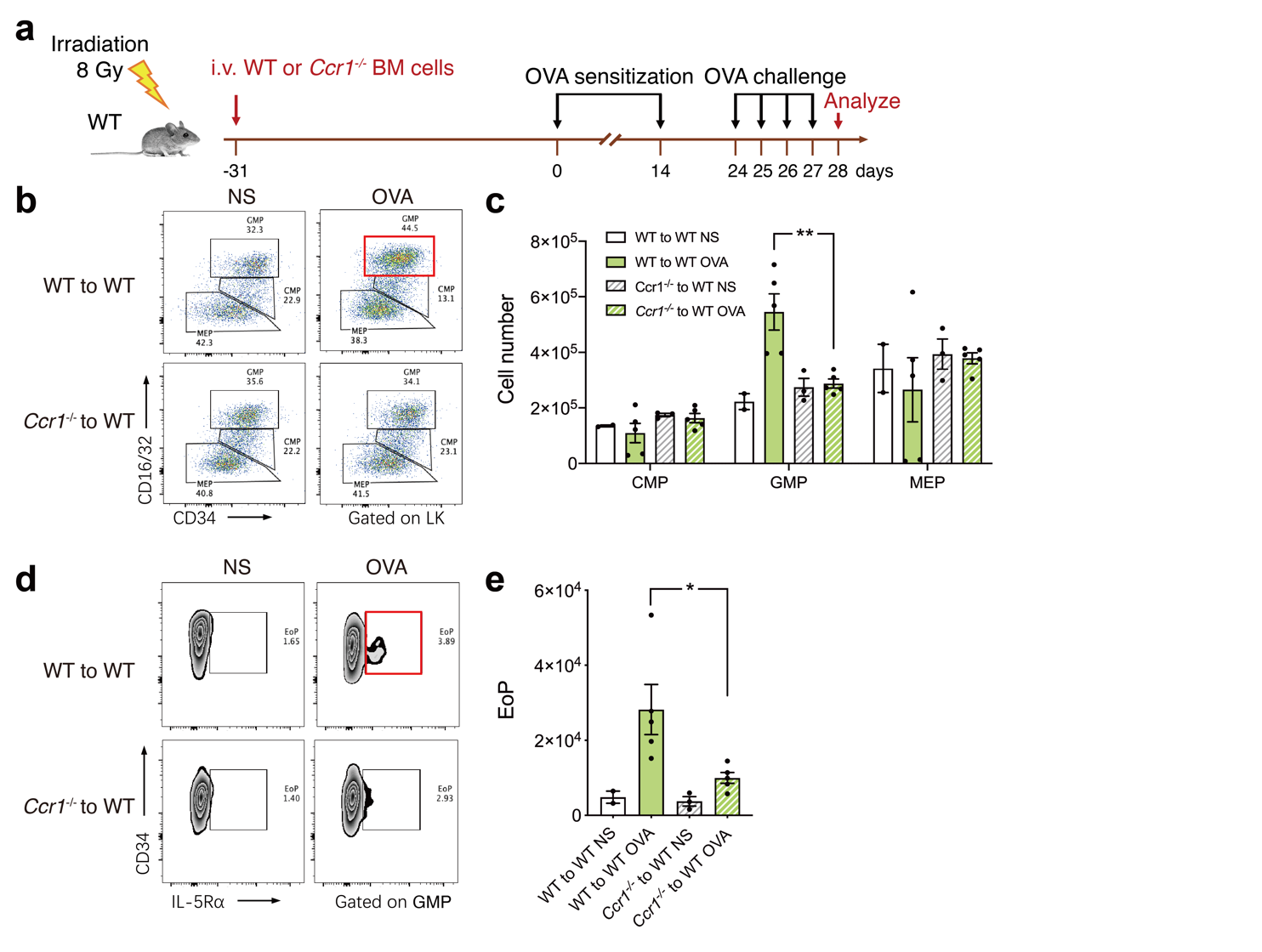


**Fig. S10** **Conditional hematopoietic CCR1-deficient mice displayed weak committed eosinophil differentiation in an OVA-challenged model.**

**a** Schematic timeline representing the allergic asthma model of mice, which received a total bone marrow transplantation from WT or *Ccr1* knockout (*Ccr1^-/-^*) mice following lethally irradiated. **b-c** Representative flow cytometric dot (**b**) and quantitative numbers (**c**) of CMP, GMP, MEP. **d-e** Representative flow cytometric dot (**d**) and quantitative numbers (**e**) of EoP of asthmatic models in **a**. Data are statistically calculated as mean ± SEM for 2-5 mice in each group. *, *P* < 0.05; **, *P* < 0.01 by one-way ANOVA with Sidak’s multiple comparisons test.

**Fig. S11**


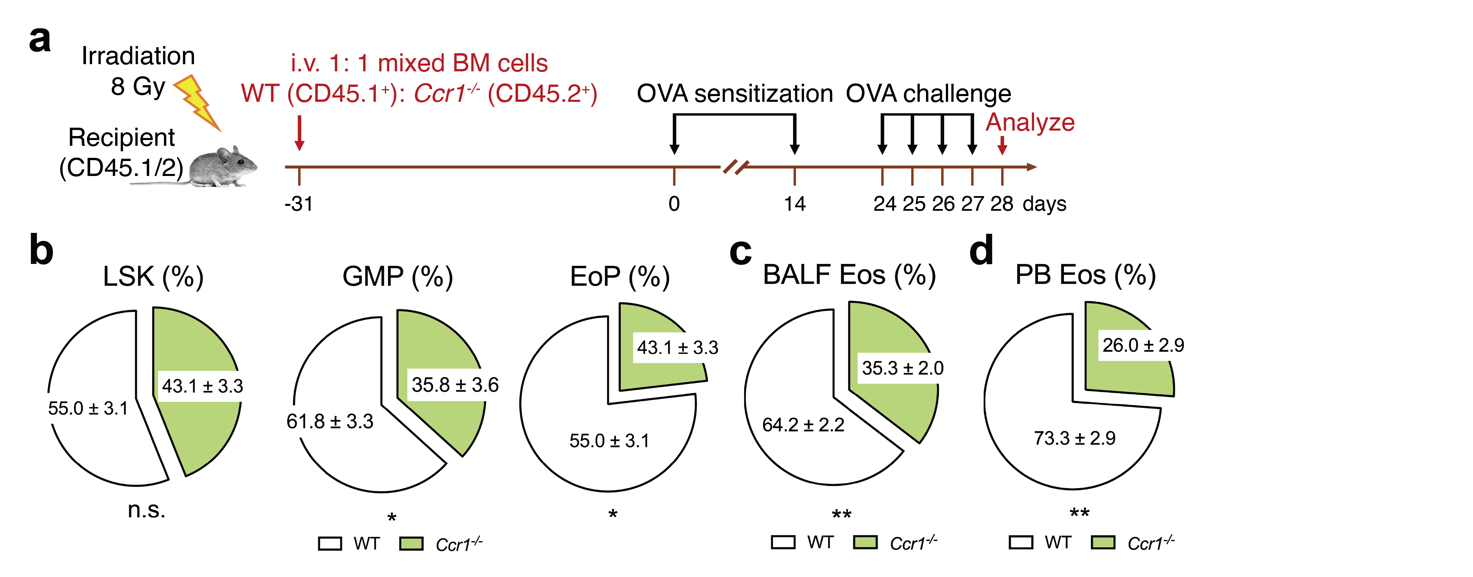


**Fig. S11** ***Ccr1^-/-^* bone marrow cells exhibited diminished eosinophilopoiesis in a chimeric mice model under OVA challenge.**

**a** Schematic timeline representing the allergic asthma models of mice, which received a competitive bone marrow transplantation with WT (CD45.1^+^) and *Ccr1^-/-^* (CD45.2^+^) bone marrow cells following lethally irradiated. **b** Percentage of WT or *Ccr1^-/-^*-derived LSK, GMP, and EoP in bone marrow of OVA mice. **c** Percentage of WT or *Ccr1^-/-^*-derived eosinophils in BALF and peripheral blood of OVA mice. Data are statistically calculated as mean ± SEM for 4 mice in each group. n.s., not significant; *, *P* < 0.05; **, *P* < 0.01 by paired *t* test.

**Fig. S12**

**
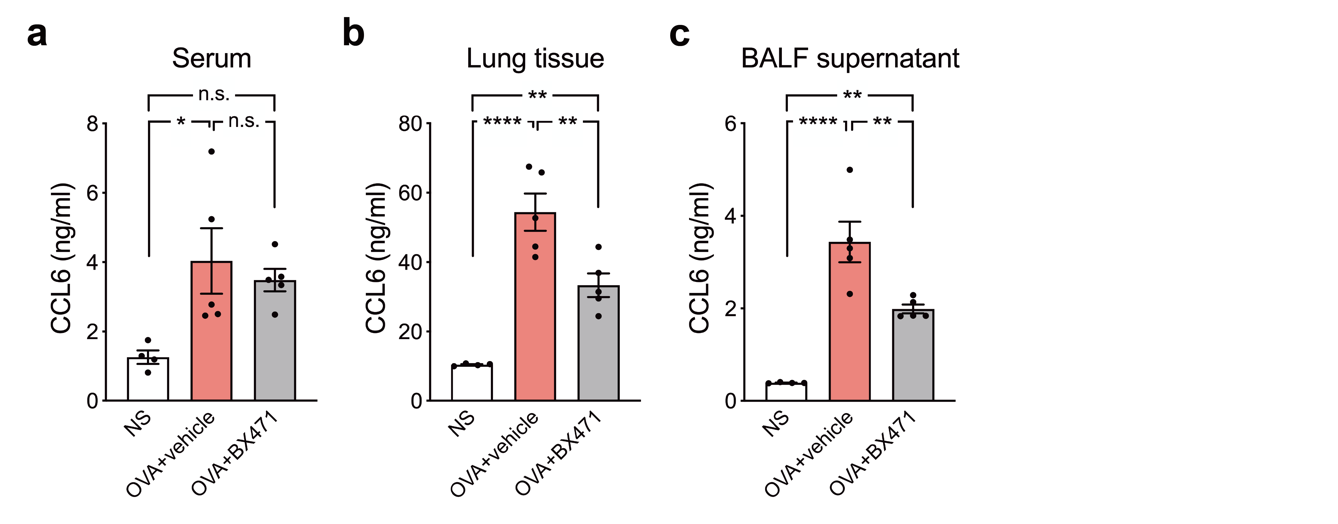
**

**Fig. S12 mCCL6 expression in BX471-treated allergic inflammatory model.**

**a-c** The concentration of mCCL6 in serum (**a**), lung tissue (**b**), and BALF supernatant (**c**) were measured by ELISA from asthmatic models with therapeutic treatment of BX471. Data are mean ± SEM for 4-5 mice per group. n.s., not significant; *, *P* < 0.05; **, *P* < 0.01; ***, *P* < 0.001; ****, *P* < 0.0001 by one-way ANOVA with Sidak’s multiple comparisons test.

**Table S1. Clinical characteristics of human subjects.**

|  | Control | Asthma |
| --- | --- | --- |
| No. | 30 | 31 |
| Age | 41.5 ± 2.2 | 46.2 ± 2.6 |
| Male (%) | 16 (53.3) | 20 (64.5) |
| Blood eosinophil (×10^3^/μl) | 0.176 ± 0.022 | 0.404 ± 0.042 |
| FEV1 (% predicated) | NA* | 66.83 ± 4.24 |
| FEV1/FVC (%) | NA | 62.84 ± 3.16 |

Data are presented as mean ± SEM or number (%). *NA, not available.

**Table S2. Complete blood counts for WT and Ccl6^-/-^ mice.**

| Leukocyte (×10^3^/μl) | WT (n=9) | Ccl6^-/-^ (n=9) | P value |
| --- | --- | --- | --- |
| WBC* | 4.206 ± 0.525 | 4.352 ± 0.486 | 0.840 |
| Lymphocyte | 3.401 ± 0.448 | 3.405 ± 0.412 | 0.996 |
| Monocyte | 0.192 ± 0.031 | 0.250 ± 0.043 | 0.293 |
| Neutrophil | 0.577 ± 0.045 | 0.595 ± 0.068 | 0.165 |
| Eosinophil | 0.133 ± 0.026 | 0.103 ± 0.012 | 0.300 |

Data are presented as mean ± SEM. *WBC, white blood cell.

**Table S3. Primer sequences for quantitative RT-PCR.**

**Primer sequences for human genes:**

| **Gene** | **Primer** | **Primer Sequence (5' to 3')** |
| --- | --- | --- |
| *hCCL23* | Forward | GTTACTGCCCTTGGATCCCAG |
| *hCCL23* | Reverse | GATCCGTGTGTCCAGCTTCAG |
| *hCCL15* | Forward | CTGACTGCTGCACCTCCTACATC |
| *hCCL15* | Reverse | GACCACTGGGTTTGGCACAGAC |
| *β-ACTIN* | Forward | CCCAGCCATGTACGTTGCTAT |
| *β-ACTIN* | Reverse | TCACCGGAGTCCATCACGAT |

**Primer sequences for mouse genes:**

| **Gene** | **Primer** | **Primer Sequence (5' to 3')** |
| --- | --- | --- |
| *Tslp* | Forward | ACGGATGGGGCTAACTTACAA |
| *Tslp* | Reverse | AGTCCTCGATTTGCTCGAACT |
| *Epx* | Forward | ACAACGGTCGAGCCCTACTGC |
| *Epx* | Reverse | TGTGCAAGGCCGTCAGTTTGGG |
| *Muc5ac* | Forward | CTGTGACATTATCCCATAAGCCC |
| *Muc5ac* | Reverse | AAGGGGTATAGCTGGCCTGA |
| *Il-4* | Forward | GGTCTCAACCCCCAGCTAGT |
| *Il-4* | Reverse | GCCGATGATCTCTCTCAAGTGAT |
| *Il-33* | Forward | ATTTCCCCGGCAAAGTTCAG |
| *Il-33* | Reverse | AACGGAGTCTCATGCAGTAGA |
| *Il-13* | Forward | CAGCCTCCCCGATACCAAAAT |
| *Il-13* | Reverse | GCGAAACAGTTGCTTTGTGTAG |
| *Il-25* | Forward | TATGAGTTGGACAGGGACTTGA |
| *Il-25* | Reverse | TGGTAAAGTGGGACGGAGTTG |
| *Ccr1* | Forward | ACTGCTGTAAGAGCCTTTGGG |
| *Ccr1* | Reverse | AGCACCAGAATCACTAGGACA |
| *β-Actin* | Forward | GGCTGTATTCCCCTCCATCG |
| *β-Actin* | Reverse | CCAGTTGGTAACAATGCCATGT |
